# Effects of intraperitoneal injection of lipopolysaccharide‐induced peripheral inflammation on dopamine neuron damage in rat midbrain

**DOI:** 10.1111/cns.13906

**Published:** 2022-07-04

**Authors:** Qiu‐Yu Yang, Xian‐Wei Li, Rong Yang, Ting‐Yang Qin, Hong Long, Shi‐Bin Zhang, Feng Zhang

**Affiliations:** ^1^ Key Laboratory of Basic Pharmacology of Ministry of Education and Joint International Research Laboratory of Ethnomedicine of Ministry of Education and Key Laboratory of Basic Pharmacology of Guizhou Province and Laboratory Animal Center Zunyi Medical University Zunyi China

**Keywords:** dopamine neuron damage, lipopolysaccharide, neuroinflammation, Parkinson's disease, peripheral inflammation

## Abstract

**Introduction:**

Current studies have documented neuroinflammation is implicated in Parkinson's disease. Recently, growing evidence indicated peripheral inflammation plays an important role in regulation of neuroinflammation and thus conferring protection against dopamine (DA) neuronal damage. However, the underlying mechanisms are not clearly illuminated.

**Methods:**

The effects of intraperitoneal injection of LPS (LPS_[i.p.]_)‐induced peripheral inflammation on substantia nigra (SN) injection of LPS (LPS_[SN]_)‐elicited DA neuronal damage in rat midbrain were investigated. Rats were intraperitoneally injected with LPS (0.5 mg/kg) daily for 4 consecutive days and then given single injection of LPS (8 μg) into SN with an interval of 0 (LPS_(i.p.)_ 0 day ± LPS_(SN)_), 30 (LPS_(i.p.)_ 30 days ± LPS_(SN)_), and 90 (LPS_(i.p.)_ 90 days ± LPS_(SN)_) days after LPS_(i.p.)_ administration.

**Results:**

LPS_(i.p.)_ increased the levels of inflammatory factors in peripheral blood in (LPS_(i.p.)_ 0 day ± LPS_(SN)_). Importantly, in (LPS_(i.p.)_ 0 day ± LPS_(SN)_) and (LPS_(i.p.)_ 30 days ± LPS_(SN)_), LPS_(i.p.)_ attenuated LPS_(SN)_‐induced DA neuronal loss in SN. Besides, LPS_(i.p.)_ reduced LPS_(SN)_‐induced microglia and astrocytes activation in SN. Furtherly, LPS_(i.p.)_ reduced pro‐inflammatory M1 microglia markers mRNA levels and increased anti‐inflammatory M2 microglia markers mRNA levels. In addition, the increased T‐cell marker expression and the decreased M1 microglia marker expression and more DA neuronal survival were discerned at the same area of rat midbrain in LPS_(SN)_‐induced DA neuronal damage 30 days after LPS_(i.p.)_ application.

**Conclusion:**

This study suggested LPS_(i.p.)_‐induced peripheral inflammation might cause T cells to infiltrate the brain to regulate microglia‐mediated neuroinflammation, thereby protecting DA neurons.

## Graphical abstract

The possible mechanisms underlying peripheral inflammation‐mediated dopamine (DA) neuroprotection. Peripheral inflammation evoked neuroprotection against LPS‐induced DA neuronal damage in rat substantia nigra, and the mechanisms might be associated with the recruitment of T cells to inhibit M1 microglial activation and the subsequent neuroinflammatory response.

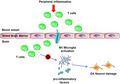

## 1 INTRODUCTION

Parkinson's disease (PD) is one of the most common neurodegenerative diseases of the central nervous system (CNS).^1^ The pathological features of PD are the selective dopamine (DA) neurons loss and the formation of α‐synuclein (α‐syn) in midbrain substantia nigra (SN). Until now, although many factors could induce PD, a great amount of evidence has documented that inflammation is closely implicated in PD.^2^, ^3^, ^4^

Neuroinflammation refers to CNS innate immune response caused by various factors, such as infection, external stimuli, and aging.^5^ Studies have confirmed that the injection of lipopolysaccharide (LPS) into the midbrain SN caused neuroinflammation and further DA neuronal damage. LPS elicits the activation of glial cells in the brain, mainly activation of microglia and astrocytes, among which microglia's immune‐regulatory effects on CNS are dominant.^6^ When CNS is not stimulated, the resident microglia and neurons maintain the balance of brain microenvironment.^7^ Upon CNS exposure to injury or inflammogen, microglia secrete a great deal of pro‐inflammatory factors, such as interleukin‐1β (IL‐1β), tumor necrosis factor‐α (TNF‐α), and interleukin‐18. These factors cause DA neuronal damage and then the damaged DA neurons release neurotoxic substances to stimulate microglia, thus leading to a malignant neuroinflammation cycle.^8^ On the other hand, microglia‐released nitric oxide (NO) can induce activation of astrocytes and the activated astrocytes also secrete pro‐inflammatory factors, such as interleukin‐2 and TNF‐α, and further cause DA neuronal damage.^9^, ^10^ Taken together, glial cells‐mediated dynamic modulation of neuroinflammation might be a key event in the degradation of PD.

In addition, activated microglia could be divided into M1 and M2 microglia according to different functions.^11^ M1 microglia not only have phagocytic function and but also secrete a large number of pro‐inflammatory factors, such as NO and interleukin‐6 (IL‐6), which aggravates DA neuronal damage.^12^ On the contrary, M2 microglia also have phagocytic function but secrete interleukin‐10 (IL‐10) and arginase‐1 (Arg‐1), which could attenuate neuroinflammatory response and further play a neuroprotective role.^13^ Therefore, the transformation of pro‐inflammatory M1 microglia and anti‐inflammatory M2 microglia might hold a promising therapeutic potential for the treatment of PD.

Growing evidence showed that peripheral inflammation played an important role in neuroinflammation recent years. For example, intraperitoneal injection of LPS (5 mg/kg) in mice caused microglia activation and DA neurons damage in SN.^14^ Moreover, extensive studies indicated that peripheral inflammation played a neuroprotective in neurodegenerative diseases. This view has been confirmed in mechanical brain injury^15^ and hypothermic circulatory block of animal models.^16^ Meanwhile, continuous intraperitoneal injection of LPS (0.3 mg/kg) in rats caused peripheral immune tolerance. These findings suggested that the peripheral immune tolerance inhibited microglia activation and inflammatory factors release in CNS, ultimately producing protection against DA neuronal damage.^17^ Furthermore, the protective effects of peripheral injection of LPS might be related to peripheral immune cells infiltrating the brain.^18^, ^19^ In PD mouse model, after intraperitoneal injection of LPS, intravenous injection of two different α‐syn pathogenic strains induced excessive activation of microglia, and further promoted the recruitment of monocytes to the brain and spinal cord to regulate neuroinflammation.^20^ Additionally, peripheral immune cells, such as T cells, B cells, and monocytes, also infiltrated the brain to affect PD progression, and the differentiation of T cells could affect the activation of microglia.^21^, ^22^ However, the mechanisms underlying this peripheral inflammation‐mediated neuroprotection are not clearly illuminated.

In this study, the effects of intraperitoneal injection of LPS‐induced peripheral inflammation on SN injection of LPS‐elicited DA neuronal damage in rat midbrain were investigated. In addition, this study also observed whether peripheral inflammation caused peripheral immune cells to infiltrate the brain and then affect the immune regulation in CNS. Together, this study aimed to explore the effects of peripheral inflammation on DA neuronal damage and its possible mechanisms.

## 2 MATERIALS AND METHODS

### 2.1 Animals and Treatment

**FIGURE 1:**
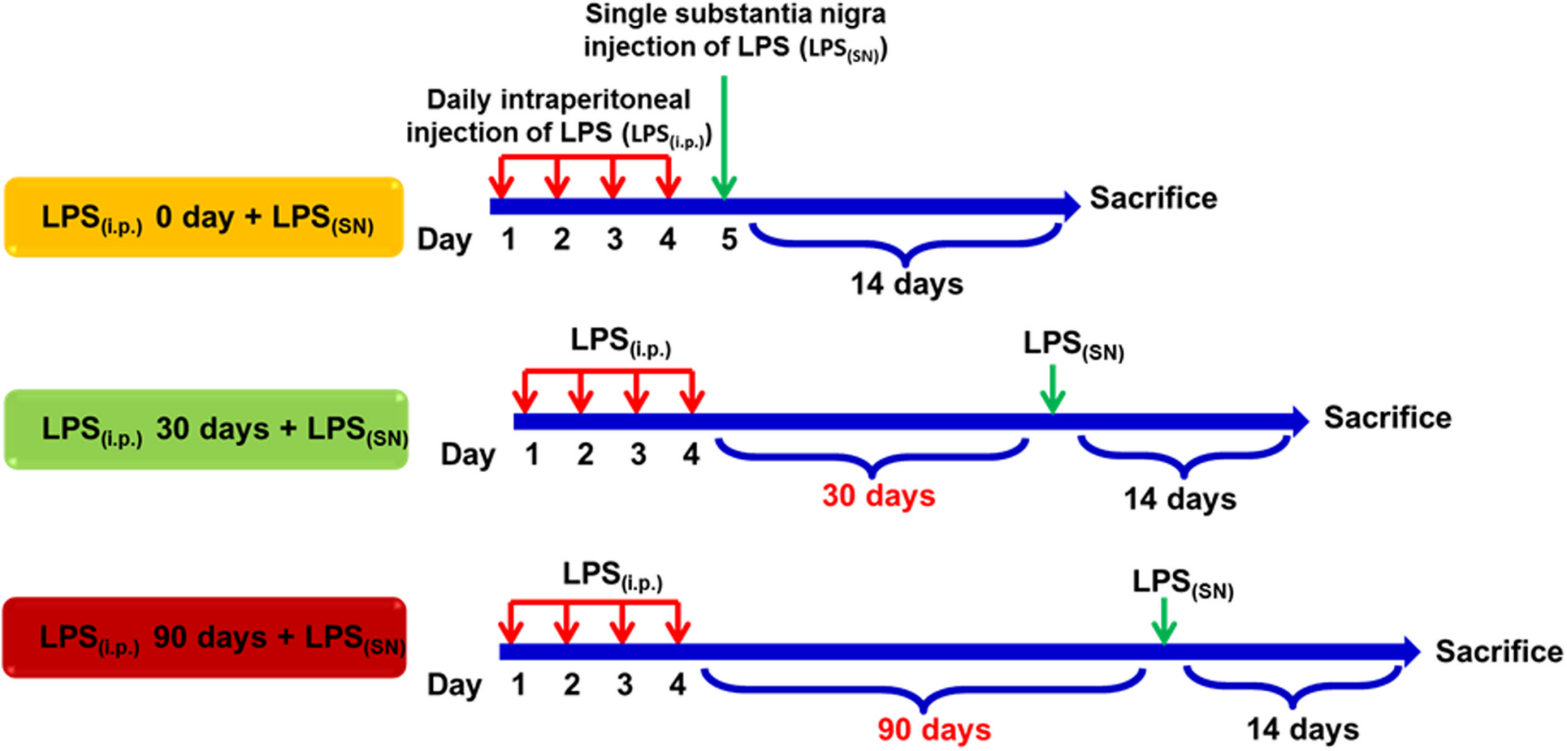
Time schedule of LPS_(i.p.)_ and LPS_(SN)_ administration

Adult male Sprague–Dawley rats (220–260 g) were purchased from Beijing HuaFuKang Bioscience Co., Ltd. Animals were housed in a temperature (19–25°C) and humidity‐controlled (40%–70%) with a 12‐h light/dark cycle and free access to autoclaved water and rat chow diet. All efforts were made to minimize their suffering. The animal data reporting followed the Animal Research: Reporting of In Vivo Experiments 2.0 guidelines^23^ and the protocols were approved by the institutional Animal Care and Use Committee at Zunyi Medical University. Rats were acclimated to their environment for 1 week before the experiments. Rats were intraperitoneally injected with LPS (0.5 mg/kg, LPS_(i.p.)_) daily for 4 consecutive days to induce peripheral inflammation and then rats received single injection of LPS (8 μg, LPS_(SN)_) into SN to induce DA neuronal damage with an interval of 0, 30 and 90 days after LPS_(i.p.)_ injection.^24^, ^25^ The detail of specific three treatment strategies, termed as (LPS_(i.p.)_ 0 day ± LPS_(SN)_), (LPS_(i.p.)_ 30 days ± LPS_(SN)_) and (LPS_(i.p.)_ 90 days ± LPS_(SN)_), was shown in Figure 1. Fourteen days after LPS_(SN)_ injection, behavioral tests were performed and then animals were sacrificed.

### 2.2 Reagents

Lipopolysaccharide (0111:B4) and anti‐dopaminergic transporter (DAT; Lot 087M4786V) antibody were from Sigma Chemical Co. Lysis Buffer and the enhanced chemiluminescence (ECL) reagent were bought from Beyotime Institute of Biotechnology. Enzyme‐linked Immunosorbent assay (ELISA) kits for TNF‐α and IL‐1β were purchased from Elabscience Biotechnology Co., Ltd. Anti‐tyrosine hydroxylase (TH; Catalog Nos. Ab41528 and Ab113), ionized calcium‐binding adapter molecule‐1 (IBA‐1, Catalog No. Ab178846), CD8α (Catalog No. Ab33786), mouse IgG H&L (TRITC), goat Anti‐Rabbit IgG H&L (TRITC), and sheep IgG H&L (Alexa Fluor 405) antibodies were bought from Abcam. Anti‐IL‐1β (Catalog No. 16806‐1‐AP), TNF‐α (Catalog No. 17590‐1‐AP), glial fibrillary acidic protein (GFAP; Catalog No. 16825‐1‐AP), β‐actin (Catalog. No. 20536‐1‐AP), rabbit IgG (Catalog. No. SA00001‐2), and mouse IgG (Catalog. No. SA00001‐1) antibodies were from Proteintech Group (Chicago, IL, USA). Biotinylated secondary antibodies and vectastain avidin–biotin complex (ABC) kits were from Vector Laboratories. PCR primers were designed using ABI Primer Express software (Applied Biosystems).

### 2.3 Rotarod test

Rotarod test was a common test used to assess animal behavior dysfunction. Before the experiment, rats were trained on the rotating rod until they stayed on the rod for at least the specified time. The starting speed of the test was 10 rpm, increasing by 5 rpm every 30 s until rats fell off the rotating rod. Rat behavior changes were analyzed by recording the time that each rat stayed on the rod.

### 2.4 Open field test

Open field test was a sensorimotor test performed to determine the general activity level, total motor activity and exploratory habits of rodent models of neurological disorders. Briefly, each rat was placed in a separate open field area, and rat behavioral parameters were recorded within 10 min. Before each round of test, the equipment was cleaned with 75% alcohol solution to avoid odor interference. After the experiment, rat total moving distance was calculated.

### 2.5 ELISA

The levels of TNF‐α and IL‐1β were measured by ELISA according to the manufacturer's instructions. The microplate reader was used to measure the absorbance at 450 nm.

### 2.6 Real‐time RT‐PCR

Total RNA was extracted by Trizol reagent and purified with RNeasy Kits. The primer sequences were inducible nitric oxide synthase (iNOS): TGGGCTGTGCAAACCTTCCG (F), TGGCTCCCATGTTGCGTTGG (R); IL‐6: CTCATTCTGTCTCGAGCCCA (F), (R); Arginase‐1 (Arg‐1): TGGAACG AAACGGGAAGGTA (F), TGTGATGCCCCAGATGACTT (R); IL‐10: GTTGCCAAGCCTTGTCAGAA (F), CAGCTTCTCTCCCAGGGAAT (R); CD8α: ACACTTCGCAAGGATGCTCT (F), GTTGCTGGTGATTGAGC AGA (R); CD4: CCCTGAACCAGAAGAAGCAC (F), GTGATCCAGGACT GGGAAGA (R); CD19: TAGGGAGTGGTCCTGTGTCC (F), TTCATAGG CCTCCCCTTCTT (R); B cell lymphoma 2 (BCL2): GGGATGCCTTT GTGGAACTA (F), CTCACTTGTGGCCCAGGTAT (R); chemokine (C‐C motif) ligand 2 (CCL2): TAGCATCCACGTGCTGTCTC (F), TGCTGCTG GTGATTCTCTTG (R); myeloperoxidase (MPO): GCCATGGGAAATA GAAGCAA (F), CCACACAGTCCAGCTGCATT (R); β‐actin: GTGCTAT GTTGCTCTAGACTTCG (F), ATGCCACAGGATTCCATACC (R). Total RNA was reversely transcribed by MuLV reverse transcriptase and Oligo‐dT primers. The SYBR green PCR Master Mix was performed for real‐time RT‐PCR analysis. The relative mRNA expression difference among different groups was measured via cycle time (Ct) values normalized with β‐actin of the same sample. The relative mRNA expressions in each group were performed and calculated.

### 2.7 Tissue preparation and immunohistochemical staining

Dopamine neurons were identified with an anti‐TH antibody. Microglia and astrocytes were recognized by anti‐IBA‐1 and GFAP antibodies, respectively. In detail, after rat motor performance was finished, rats were anesthetized and transcardially perfused with cold PBS before 4% formaldehyde was used to fix. Then, brains were removed and post‐fixed with 4% formaldehyde for 48 h. Next, formaldehyde (4%) was replaced by 30% sucrose solution at 4°C until tissues sank. Brains were cut into 35 μm using a horizontal sliding microtome and brain slices were placed in plate containing PBS and stored at 4°C. In brief, the brain slices were first treated with 0.3% Triton X‐100 for 20 min and re‐acted with 3% H_2_O_2_ for 15 min, and then blocked with goat serum for 30 min. The slices were incubated overnight at 4°C with primary rabbit anti‐TH (1:500), rabbit anti‐IBA‐1 (1:500) and rabbit anti‐GFAP (1:500) antibodies. Slides were incubated with biotinylated secondary antibody at 37°C for 25 min and visualized through DAB kit. Quantification of TH‐positive neurons were determined by visually counting the number of TH‐positive neuronal cell bodies. Activation of microglia and astrocytes was detected by the fluorescence intensity analysis of IBA‐1‐positive microglia and GFAP‐positive astrocytes, respectively. Results were obtained from the average. The mean value was deduced by averaging the counts of six sections of each rat brain.

### 2.8 Immunofluorescence staining

Brains were cut into 35 μm using a horizontal sliding microtome and brain slices were placed in plate containing PBS and stored at 4°C. Briefly, the brain slices were first treated with 0.3% Triton X‐100 for 30 min and blocked with goat serum. The slices were incubated overnight at 4°C with sheep anti‐TH (1:200), mouse anti‐CD8α (1:200) and rabbit anti‐iNOS (1:200) antibodies. Then, brain tissues were incubated in fluorescent‐conjugated secondary antibody for 30 min. Digital images of TH‐positive neurons, CD8α‐positive T cell and iNOS‐positive microglia in rat SN were acquired by an Olympus microscope (Olympus) via an attached Polaroid digital microscope camera (Polaroid). Quantification of these positive cells was detected by the fluorescence intensity analysis, respectively. Results were obtained from the average. The mean value was deduced by averaging the counts of six sections for each rat brain.

### 2.9 Western blot analysis

After rats were deeply anesthetized, brains were quickly dissected and separated on ice and the separated midbrain and striatum tissues were frozen at −80°C. The frozen tissue was homogenized with lysis buffer (including protease inhibitor and phosphate protease inhibitor) and cracked on ice. The supernatant was collected after centrifugation for 10 min at 4°C. Protein concentrations were detected by BCA assay kit. An equal amount of protein for each sample was loaded onto a sodium dodecyl sulfate polyacrylamide electrophoresis gel (10%). Proteins separated by gel electrophoresis were electro transferred onto a 0.45 mm polvinylidene difluoride membranes. The membranes were blocked with 5% skimmed milk, and then incubated at 4°C overnight with primary rabbit anti‐TH (1:2000), rabbit anti‐DAT (1:1000), rabbit anti‐IL‐1β (1:1000), rabbit anti‐TNF‐α (1:1000), rabbit anti‐IBA‐1 (1:1000), rabbit anti‐GFAP (1:2000), and rabbit anti‐β‐actin (1:2000) antibodies. After washing, bound antibodies were detected with HRP‐conjugated secondary anti‐rabbit antibody (1:3000). The blots were developed with ECL kit and quantified with the software Quantity one.

### 2.10 Statistical analysis

Data were expressed as mean ± standard error of the mean (SEM). Statistical significance was analyzed by one‐way analysis of variance (ANOVA) using GraphPad Prism software (GraphPad Software Inc.). When ANOVA showed significant difference, pairwise comparisons between means were accessed by Bonferroni's post hoc *t‐*test with correction. All data were tested for normality. Data that did not exhibit a normal/Gaussian distribution were analyzed by non‐parametric equivalents. A value of *p* < 0.05 was considered statistically significant.

## 3 RESULTS

### 3.1 Effects of LPS_(i.p.)_ followed by LPS_(SN)_ on the levels of inflammatory factors in rat peripheral blood

**FIGURE 2:**
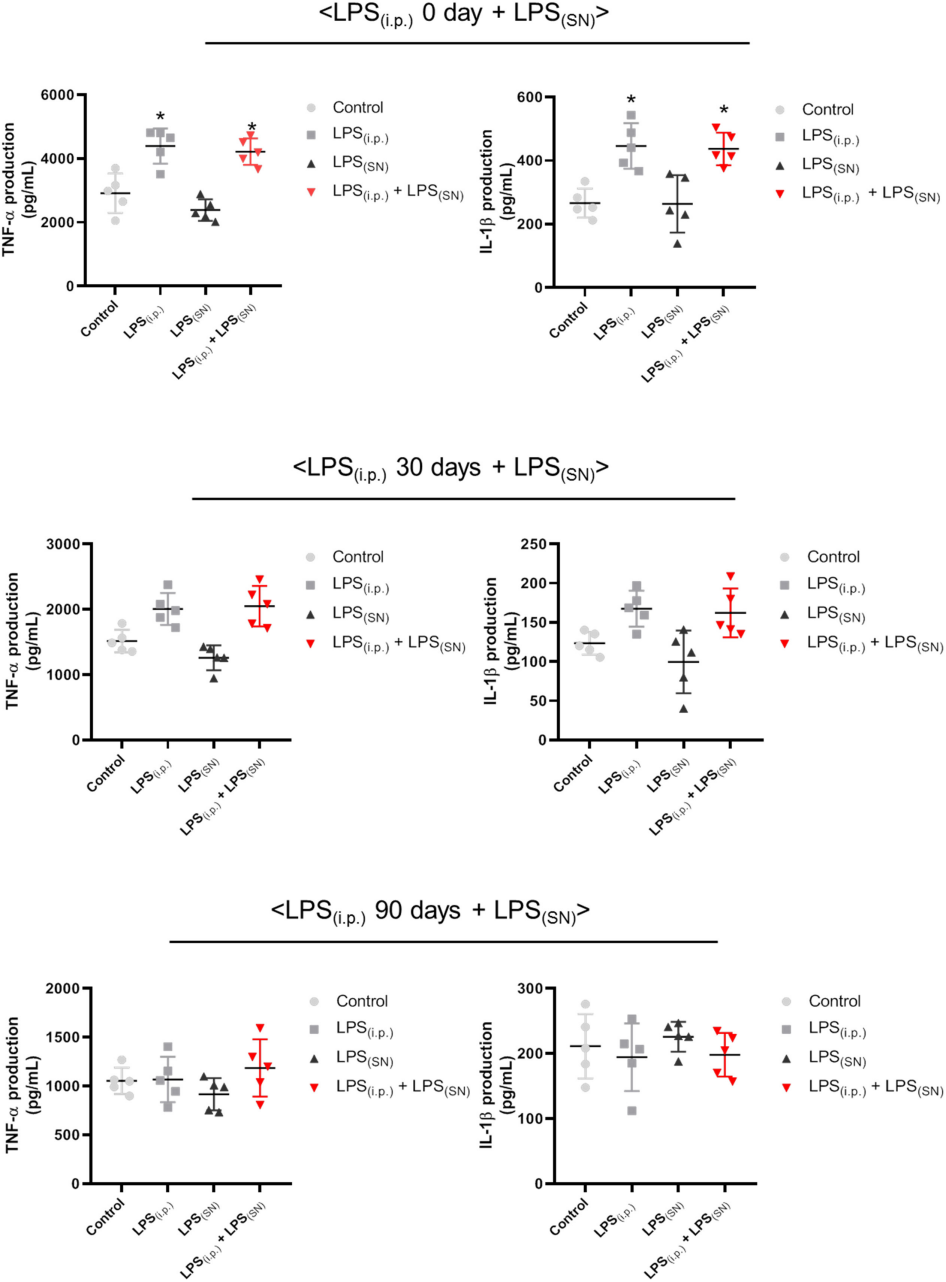
Effects of LPS_(i.p.)_ followed by LPS_(SN)_ on the levels of inflammatory factors in rat peripheral blood. Rats were intraperitoneally injected with LPS (0.5 mg/kg, LPS_(i.p.)_) daily for 4 consecutive days to induce peripheral inflammation model and then rats were single injected with LPS (8 μg, LPS_(SN)_) into SN to induce DA neuron damage with an interval of 0, 30 and 90 days after LPS_(i.p.)_ administration. Fourteen days after LPS_(SN)_ injection, the levels of inflammatory cytokines, such as TNF‐α and IL‐1β, in rat peripheral blood on these three treatment experiments of (LPS_(i.p.)_ 0 day ± LPS_(SN)_), (LPS_(i.p.)_ 30 days ± LPS_(SN)_), and (LPS_(i.p.)_ 90 days ± LPS_(SN)_) were detected by ELISA. Data were represented as mean ± SEM from five rats. *p < 0.05 compared with control group

Peripheral inflammation in rats was induced by intraperitoneal injection of LPS. Rat peripheral blood was collected and the levels of inflammatory factors, such as TNF‐α and IL‐1β, in peripheral blood were detected. First, as shown in Figure 2, in the experiment of (LPS_(i.p.)_ 0 day ± LPS_(SN)_): compared with control group, the elevated levels of inflammatory factors were indicated in LPS_(i.p.)_ and LPS_(i.p.)_ + LPS_(SN)_ groups but not in LPS_(SN)_ group. Then, in the experiments of (LPS_(i.p.)_ 30 days ± LPS_(SN)_) and (LPS_(i.p.)_ 90 days ± LPS_(SN)_), compared with control group, no significant changes of inflammatory factors levels were shown in LPS_(i.p.)_, LPS_(SN)_ and LPS_(i.p.)_ + LPS_(SN)_ groups. These results suggested that intraperitoneal injection of LPS increased the level of inflammatory factors in peripheral blood and caused acute peripheral inflammation in rats.

### 3.2 Effects of LPS_(i.p.)_ on LPS_(SN)_‐induced DA neuronal damage in rat SN

**FIGURE 3:**
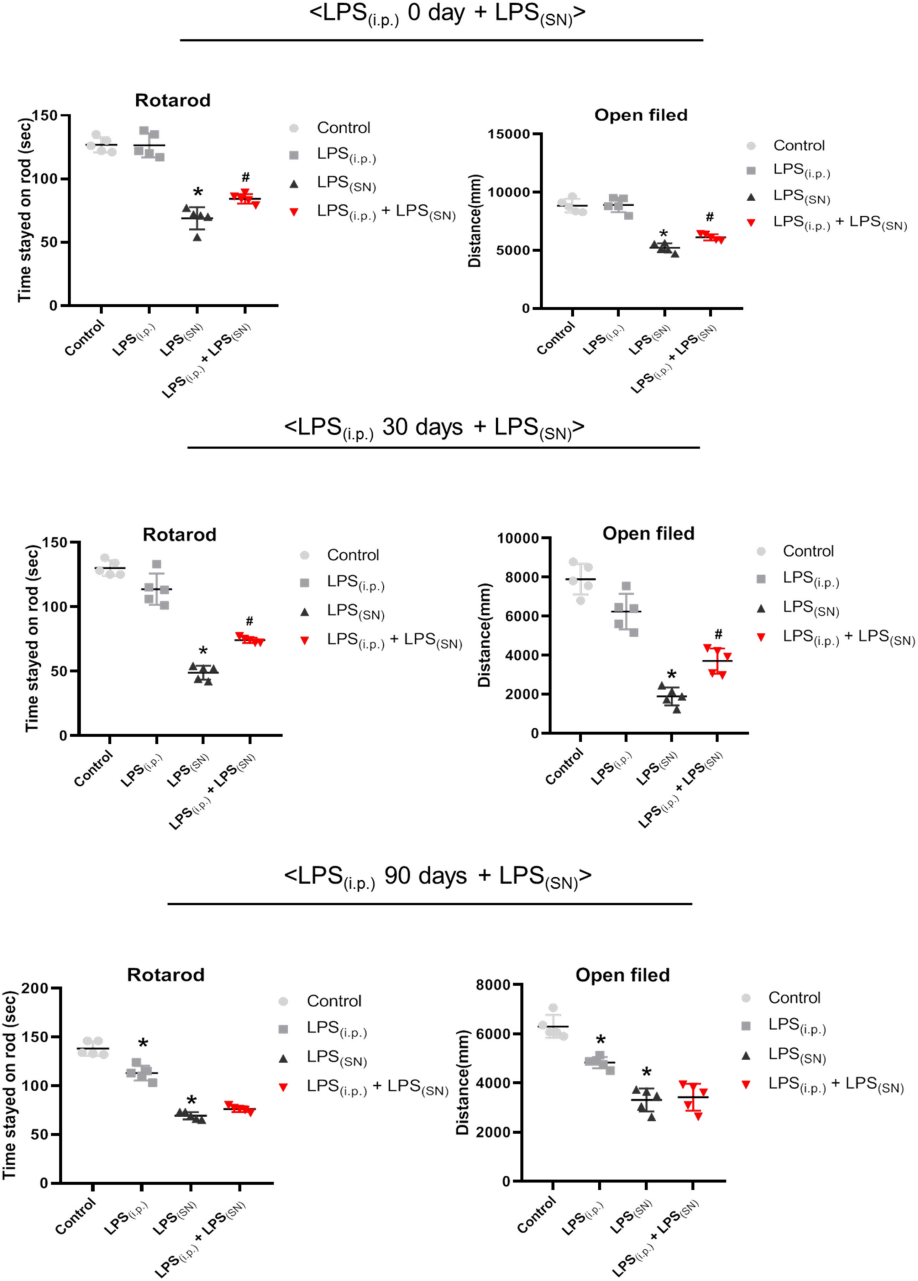
Effects of LPS_(i.p.)_ on LPS_(SN)_‐induced rat behavior dysfunctions. Rat behavior changes were analyzed by rotarod test and open field test in three treatment experiments of (LPS_(i.p.)_ 0 day ± LPS_(SN)_), (LPS_(i.p.)_ 30 days ± LPS_(SN)_), and (LPS_(i.p.)_ 90 days ± LPS_(SN)_). Data were represented as mean ± SEM from five rats. *p < 0.05 compared with control group; ^#^ *p* < 0.05 compared with LPS_(SN)_ group

First, as shown in Figure 3, in the experiment of (LPS_(i.p.)_ 0 day ± LPS_(SN)_), compared with control group, LPS_(SN)_ decreased the time rat stayed on the rod (rotarod test) and moving distance (open field test). Compared with LPS_(SN)_ group_,_ LPS_(i.p.)_ attenuated LPS_(SN)_‐induced decrease in time rat remained on the rod and movement distance. Similar results were discerned in the experiment of (LPS_(i.p.)_ 30 days ± LPS_(SN)_). However, in the experiment of (LPS_(i.p.)_ 90 days ± LPS_(SN)_), the decreased time rat stayed on the rod and moving distance were indicated in both LPS_(i.p.)_ and LPS_(SN)_ groups, whereas LPS_(i.p.)_ did not ameliorate LPS_(SN)_‐induced these decreases.

**FIGURE 4:**
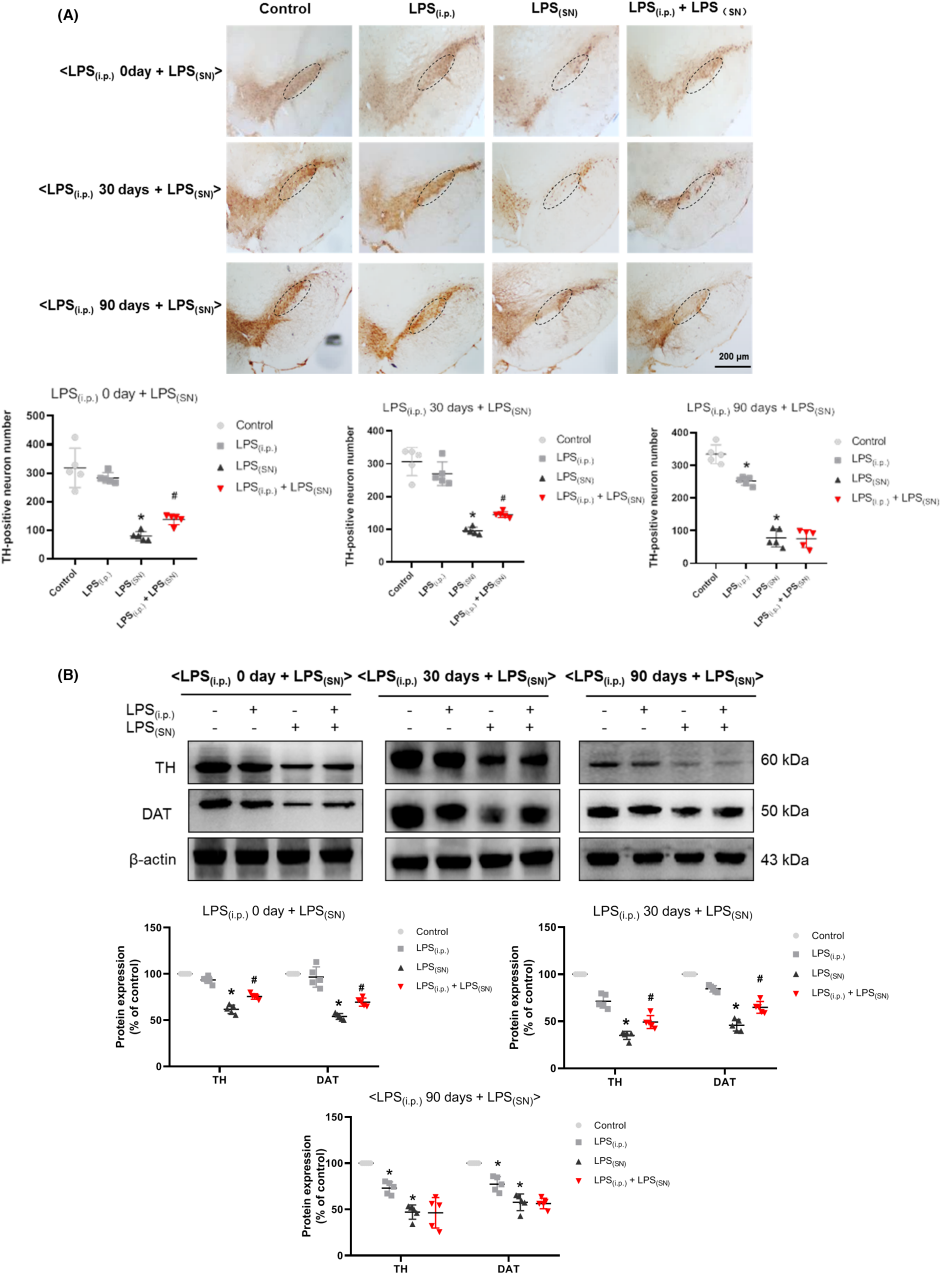
Effects of LPS_(i.p.)_ on LPS_(SN)_‐induced DA neuronal damage in rat SN. In three treatment experiments of (LPS_(i.p.)_ 0 day ± LPS_(SN)_), (LPS_(i.p.)_ 30 days ± LPS_(SN)_), and (LPS_(i.p.)_ 90 days ± LPS_(SN)_), DA neuronal number was counted via immunostaining with an anti‐TH antibody (A). The “ellipse” presented the area of SN. The protein expressions of TH and DAT (DA transporter) were determined by western blot assay (B). Data were represented as mean ± SEM from five rats. *p < 0.05 compared with control group; ^#^ *p* < 0.05 compared with LPS_(SN)_ group

Then, the effects of LPS_(i.p.)_ on LPS_(SN)_‐induced DA neurons damage were investigated. As shown in Figure 4A, DA neuron quantification results demonstrated that LPS_(i.p.)_ attenuated LPS_(SN)_‐induced DA neuronal loss in the experiments of (LPS_(i.p.)_ 0 day ± LPS_(SN)_) and (LPS_(i.p.)_ 30 days ± LPS_(SN)_). In contrast, in the experiment of (LPS_(i.p.)_ 90 days ± LPS_(SN)_), LPS_(i.p.)_ and LPS_(SN)_ caused DA neuronal loss and no neuroprotective effects of LPS_(i.p.)_ on LPS_(SN)_‐induced DA neuronal damage were exhibited. Protein expressions of TH and DAT results (Figure 4B) were consistent with DA neuron quantification.

### 3.3 Effects of LPS_(i.p.)_ on LPS_(SN)_‐induced activation of glial cells in rat SN

**FIGURE 5:**
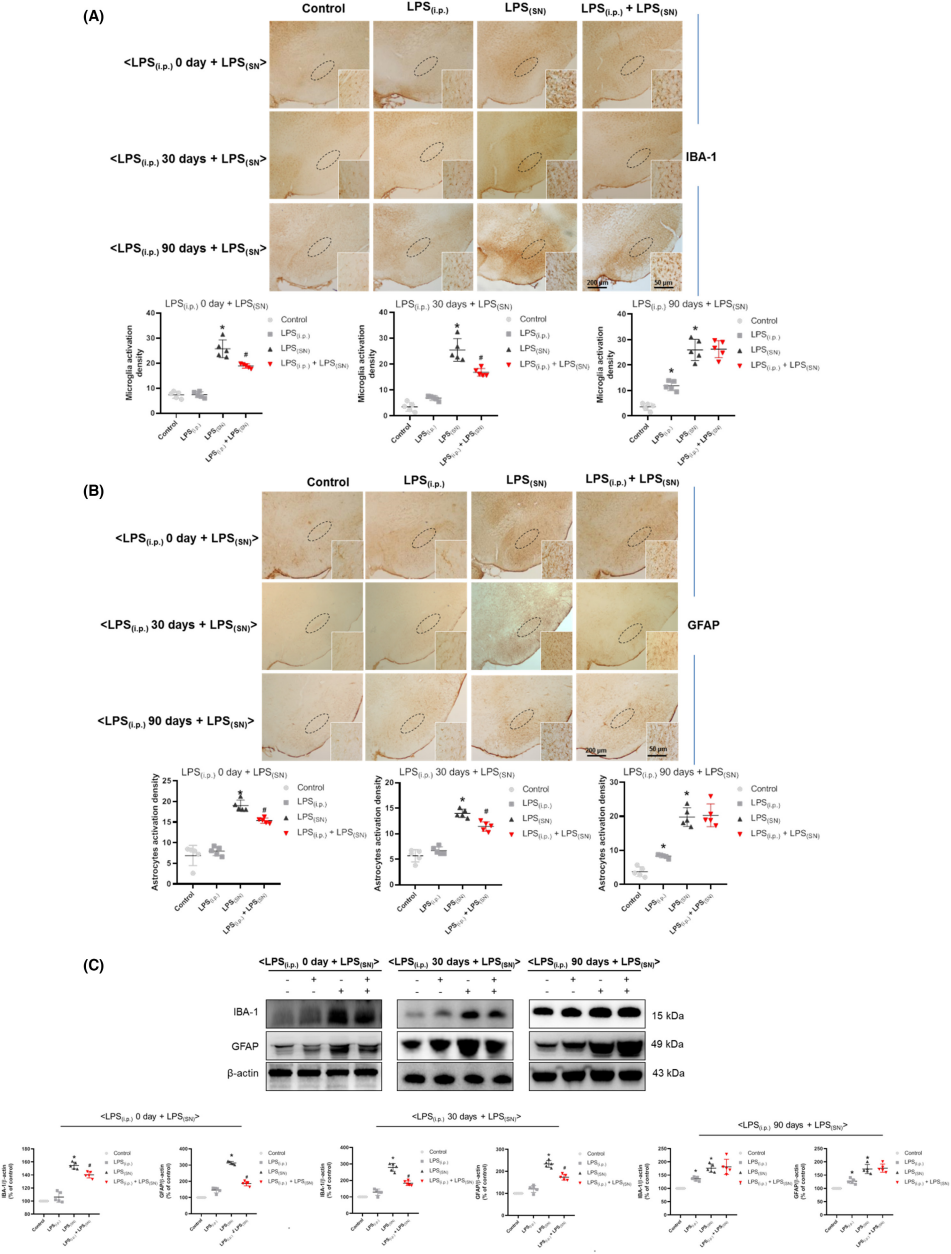
Effects of LPS_(i.p.)_ on LPS_(SN)_‐induced activation of glial cells in rat SN. Within three treatment experiments of (LPS_(i.p.)_ 0 day ± LPS_(SN)_), (LPS_(i.p.)_ 30 days ± LPS_(SN)_), and (LPS_(i.p.)_ 90 days ± LPS_(SN)_), the activated microglia and astrocytes in the SN of rat midbrain were detected by immunocytochemical staining with anti‐IBA‐1 and GFAP antibodies, respectively. The density of activated microglia (A) and astrocytes (B) was recorded. The “ellipse” presented the area of SN. The protein expressions of IBA‐1 and GFAP were detected by western blot assay (C). Data were represented as mean ± SEM from five rats. *p < 0.05 compared with control group; ^#^ *p* < 0.05 compared with LPS_(SN)_ group

Next, the effects of LPS_(i.p.)_ on LPS_(SN)_‐induced activation of microglia and astrocytes in rat SN were explored. As shown in Figure 5A,B, LPS_(i.p.)_ reduced LPS_(SN)_‐induced microglia and astrocytes activation in the experiments of (LPS_(i.p.)_ 0 day ± LPS_(SN)_) and (LPS_(i.p.)_ 30 days ± LPS_(SN)_). However, in the experiment of (LPS_(i.p.)_ 90 days ± LPS_(SN)_), LPS_(i.p.)_ and LPS_(SN)_ induced microglia and astrocytes activation and no inhibitory effects of LPS_(i.p.)_ on LPS_(SN)_‐induced microglia and astrocytes activation were present. Similar results were emerged in IBA‐1 and GFAP protein expressions detection (Figure 5C).

### 3.4 Effects of LPS_(i.p.)_ on LPS_(SN)_‐induced release of cytokines in rat SN

**FIGURE 6:**
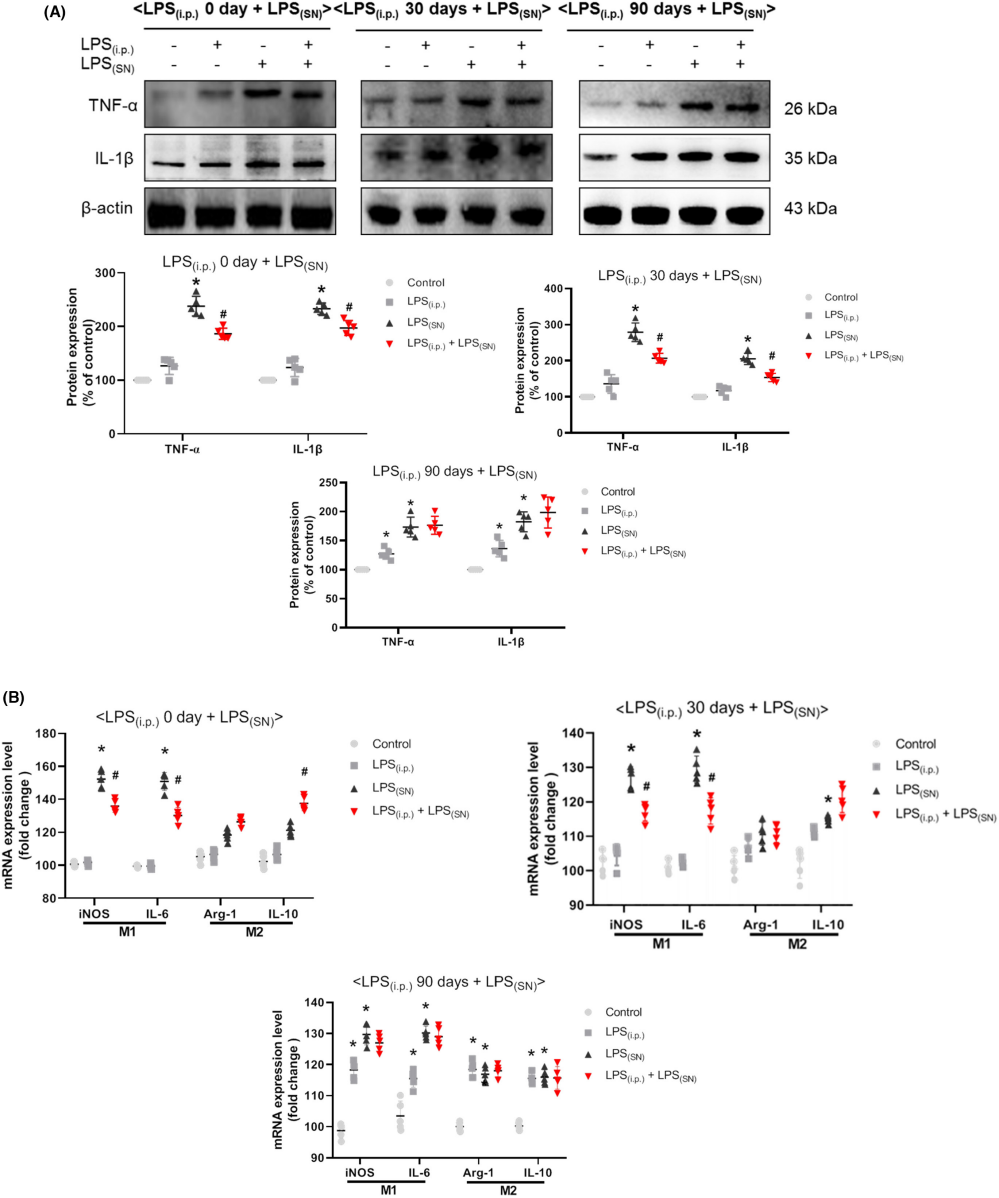
Effects of LPS_(i.p.)_ on LPS_(SN)_‐induced release of cytokines in rat SN. The protein levels of pro‐inflammatory mediators, such as TNF‐α and IL‐1β, in rat midbrain were detected by western blot assay (A). The mRNA levels of pro‐inflammatory M1 phenotype microglia marker (iNOS and IL‐6) and anti‐inflammatory M2 phenotype microglia marker (Arg‐1 and IL‐10) in rat midbrain were measured by real‐time RT‐PCR (B). Data were represented as mean ± SEM from five rats. *p < 0.05 compared with control group; ^#^ *p* < 0.05 compared with LPS_(SN)_ group

We further investigated the effects of LPS_(i.p.)_ on LPS_(SN)_‐induced release of cytokines in rat SN. As shown in Figure 6A, LPS_(i.p.)_ decreased LPS_(SN)_‐induced increase of TNF‐α and IL‐1β protein expressions in the experiments of (LPS_(i.p.)_ 0 day ± LPS_(SN)_) and (LPS_(i.p.)_ 30 days ± LPS_(SN)_) but not in the experiment of (LPS_(i.p.)_ 90 days ± LPS_(SN)_).

Since microglia are the main innate immune cells of CNS, the effects of LPS_(i.p.)_ on pro‐inflammatory M1 microglia and anti‐inflammatory M2 microglia after LPS_(SN)_ application were evaluated. As shown in Figure 6B, in the experiment of (LPS_(i.p.)_ 0 day ± LPS_(SN)_), LPS_(i.p.)_ reduced LPS_(SN)_‐induced increase of M1 microglia markers (iNOS and IL‐6) mRNA levels. Besides, M2 microglia marker (IL‐10) mRNA level was increased in LPS_(i.p.)_ + LPS_(SN)_ group. Similar results were exhibited in the experiments of (LPS_(i.p.)_ 30 days ± LPS_(SN)_) and (LPS_(i.p.)_ 90 days ± LPS_(SN)_).

### 3.5 Effects of LPS_(i.p.)_ combined with LPS_(SN)_ on immune cells in rat midbrain

**FIGURE 7:**
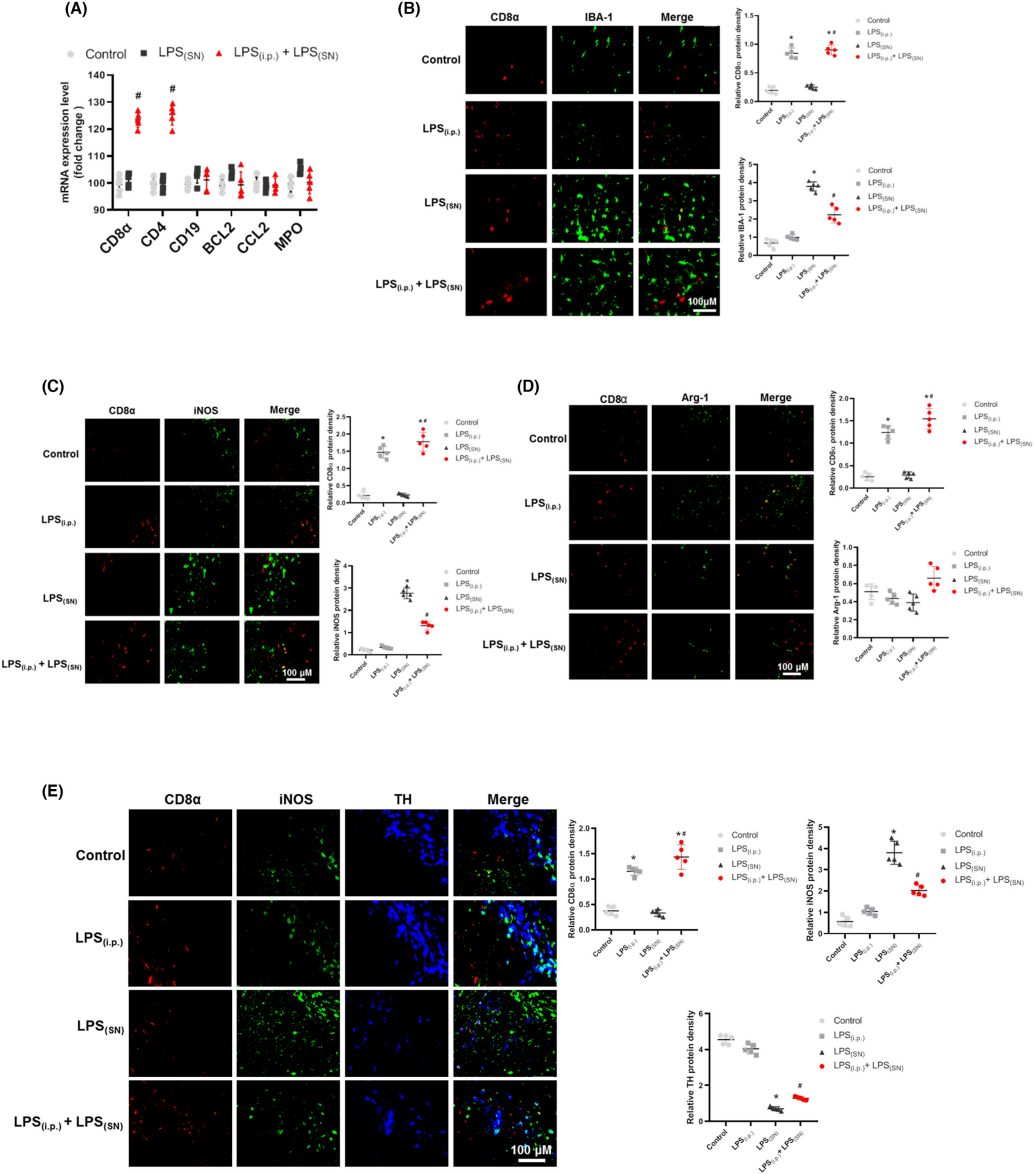
Effects of LPS_(i.p.)_ combined with LPS_(SN)_ on immune cells in rat midbrain. In (LPS_(i.p.)_ 30 days ± LPS_(SN)_) experiment, the levels of T‐cell marker (CD4 and CD8α), B‐cell marker (CD19 and BCL2) and monocyte marker (CCL‐2 and MPO) in rat midbrain were detected by real‐time RT‐PCR (A). Immunofluorescence co‐localization of T cell and M1 microglia with anti‐CD8α (red) and IBA‐1 (green) antibodies in rat midbrain was determined (B). Immunofluorescence co‐localization of T cell and M1 microglia with anti‐CD8α (red) and iNOS (green) antibodies was performed (C). Immunofluorescence co‐localization of T cell and M2 microglia with anti‐CD8α (red) and Arg‐1 (green) antibodies was determined (D). Immunofluorescence co‐localization of T cell, M1 microglia, and DA neuron with anti‐CD8α (red), iNOS (green), and TH (blue) antibodies was performed (E). Data were represented as mean ± SEM from five rats. *p < 0.05 compared with control group; ^#^ *p* < 0.05 compared with LPS_(SN)_ group

Studies have confirmed that peripheral immune cells could infiltrate the CNS. Therefore, we first detected the mRNA expressions of T‐cell markers (CD8α and CD4), B‐cell markers (CD19 and BCL2), and monocyte markers (CCL2 and MPO) in rat midbrain. As shown in Figure 7A, in the experiment of (LPS_(i.p.)_ 30 days ± LPS_(SN)_), compared with control and LPS_(SN)_ groups, the mRNA expressions of CD8α and CD4 in LPS_(i.p.)_ + LPS_(SN)_ group were increased and no changes of the other genes mRNA expressions were discerned. Then, co‐localization of CD8α and IBA‐1 protein expressions in rat SN was detected. As shown in Figure 7B, compared with LPS_(SN)_ group, IBA‐1 expression was reduced in LPS_(i.p.)_ + LPS_(SN)_ group, in which CD8α expression was increased. These results suggested that peripheral inflammation induced T cells to infiltrate CNS to further regulate the immune response in midbrain. To confirm whether T cells influenced microglia phenotype changes in CNS, the co‐localization of CD8α and M1 microglia marker iNOS or M2 microglia marker Arg‐1 protein expressions was determined. As shown in Figure 7C, compared with LPS_(SN)_ group, the decreased iNOS expression and meantime the increased CD8α expression were indicated in LPS_(i.p.)_ + LPS_(SN)_ group. However, no significant effects of LPS_(i.p.)_ + LPS_(SN)_ on Arg‐1 protein expression were detected (Figure 7D). These findings implied that T cells might be involved in the regulation of activation of M1 microglia induced by LPS_(SN)_. Finally, to further confirm the role of T cells on LPS_(i.p.)_‐attenuated DA neuronal damage induced by LPS_(SN)_, co‐localization of CD8α, iNOS and TH protein expressions was performed. As shown in Figure 7E, compared with LPS_(SN)_ group, the increased TH and CD8α protein expressions and the decreased iNOS expression at the same area of rat midbrain in LPS_(i.p.)_ + LPS_(SN)_ group were exhibited.

## 4 DISCUSSION

This study investigated the effects of peripheral inflammation on DA neuronal damage in rat midbrain and further explored how peripheral inflammation affected the neuroinflammatory response and then generated DA neuroprotection. First, LPS_(i.p.)_ increased the levels of inflammatory factors in peripheral blood in (LPS_(i.p.)_ 0 day ± LPS_(SN)_). Importantly, in (LPS_(i.p.)_ 0 day ± LPS_(SN)_) and (LPS_(i.p.)_ 30 days ± LPS_(SN)_), LPS_(i.p.)_ attenuated LPS_(SN)_‐induced DA neuronal loss in SN. Besides, LPS_(i.p.)_ reduced LPS_(SN)_‐induced microglia and astrocytes activation in SN. Furtherly, LPS_(i.p.)_ reduced LPS_(SN)_‐induced increase of M1 microglia markers (iNOS and IL‐6) mRNA levels and increased M2 microglia markers (IL‐10) mRNA levels. In addition, the increased T‐cell marker (CD8α) expression and the decreased M1 microglia marker (iNOS) expression and more DA neuronal survival were shown at the same area of rat midbrain in LPS_(SN)_‐induced DA neuronal damage 30 days after LPS_(i.p.)_ application. Taken together, this study suggested LPS_(i.p.)_‐induced peripheral inflammation might cause T cells to infiltrate the brain to regulate microglia‐mediated neuroinflammation, thereby protecting DA neurons.

At present, neuroinflammation has been confirmed to be closely involved in the development of PD.^26^ Neuroinflammation is mainly mediated by activated microglia, accompanied secretion of pro‐inflammatory factors.^27^, ^28^ When CNS is stimulated, microglia can elicit neuroinflammation. However, existing studies indicated that peripheral inflammation also caused neuroinflammation and released inflammatory factors to aggravate neuroinflammation. On the other hand, chronic neuroinflammation caused pro‐inflammatory M1 microglia to transform to anti‐inflammatory M2 microglia to protect neurons.^29^, ^30^ This study found that in the experiments of (LPS_(i.p.)_ 0 day ± LPS_(SN)_) and (LPS_(i.p.)_ 30 days ± LPS_(SN)_) but not in LPS_(i.p.)_ 90 days ± LPS_(SN)_, LPS_(i.p.)_ attenuated LPS_(SN)_‐induced M1 microglia activation. It might be speculated that chronic neuroinflammation occurred 90 days after intraperitoneal injection of LPS, which in turn led to the increased activation of M1 microglia and then caused DA neuronal damage.

More and more evidence demonstrated that the neuroprotection mediated by peripheral LPS preconditioning had been verified in various neurological disorders.^31^, ^32^, ^33^ For example, studies reported that peripheral inflammation modulated the cellular inflammatory response after cerebral ischemia, thereby producing neuroprotection in brain.^34^ Moreover, continuous intraperitoneal injection of low‐dose LPS‐induced peripheral blood mononuclear cell tolerance could reduce the secretion of inflammatory factors in SN. The mechanism might be related to regulation of CD200/CD200R signaling pathway.^35^ Similar to this publication, the present study found that in the experiment of (LPS_(i.p.)_ 0 day ± LPS_(SN)_), the increased level of inflammatory factors in rat's peripheral blood and the decreased expression of inflammatory factors in rat midbrain were shown in LPS_(i.p.)_ + LPS_(SN)_ group. These findings suggested that peripheral immune tolerance might participate in LPS_(i.p.)_‐attenuated DA neuronal damage induced by LPS_(SN)_. The underling mechanisms, including the role of immune tolerance involved in this neuroprotection, warrant further investigation.

Additionally, studies demonstrated that peripheral inflammation caused the destruction of the blood–brain barrier and then T cells entered the brain to inhibit neuroinflammatory response and finally affected the progress of PD.^36^, ^37^, ^38^ CD4 and CD8 are mature T‐cell surface markers. Previous studies revealed that α‐syn could induce autoimmunity of CD4^+^‐ and CD8^+^‐T cells to delay the process of PD.^39^ In this study, by measuring peripheral immune cell T cells, B cells and monocyte surface markers expressions in rat midbrain, the expressions of CD4 and CD8α were increased in LPS_(i.p.)_ + LPS_(SN)_ group, whereas no apparent changes of B cells and monocytes were discerned. These findings supported that peripheral inflammation might participate in the inflammatory response via inducing T cells to infiltrate the brain.

Furthermore, it has been confirmed that T cells stimulated the activation of microglia in CNS.^40^ This study detected the co‐localization of T cells and microglia. Results showed that in LPS_(i.p.)_ + LPS_(SN)_ group, the decreased activation of microglia in the area was exhibited, where T‐cell expression was increased, suggesting that T cells could suppress the activation of microglia in midbrain. Since the activated T cells regulated the activation of microglia phenotypes,^41^ the co‐localization of CD8α (T cells marker) with iNOS (M1 microglia marker) or Arg‐1 (M2 microglia marker) was further detected. Results showed that compared with LPS_(SN)_ group, in LPS_(i.p.)_ + LPS_(SN)_ group, iNOS expression was decreased in the area where T‐cell expression was increased. Besides, T‐cell CD8 signaling pathway was confirmed to regulate the activation of M1 microglia in animal models of cerebral ischemia.^42^ To further confirm the role of T cells on peripheral inflammation‐mediated DA neuroprotection, the co‐localization of CD8α, iNOS, and TH (DA neuron marker) in rat midbrain was evaluated. Data showed that in LPS_(i.p.)_ + LPS_(SN)_ group, iNOS expression was decreased in the area where the expression of CD8α was increased and the damaged DA neurons were protected. Collectively, this study suggested that LPS_(i.p.)_‐induced peripheral inflammation might cause T cells to infiltrate the brain to regulate microglia‐mediated inflammation, thereby protecting DA neurons. However, how peripheral inflammation elicited T cells to enter the brain needs further exploration.

## 5 CONCLUSIONS

This study indicated that peripheral inflammation evoked neuroprotection against LPS‐induce DA neuronal damage in rat SN, and the mechanisms might be associated with the recruitment of T cells to inhibit M1 microglial activation and the subsequent neuroinflammatory response.

## AUTHORS’ CONTRIBUTION

FZ conceived and designed the experiments. QYY participated in the experiments performance and XWL, RY, TYQ, HL, SBZ and FZ finished data analysis. QYY wrote this manuscript and FZ revised this article. All authors read and approved the final manuscript.

## CONFLICT OF INTEREST

The authors declared no any conflict of interest.

## Supporting information


Appendix S1

## Data Availability

Data that support the findings of the present study were available from the corresponding author upon reasonable request.
