# Supplementary material for: Effects of intraperitoneal injection of lipopolysaccharide‐induced peripheral inflammation on dopamine neuron damage in rat midbrain
Source: CNS Neurosci Ther. 2022 Jul 4;28(10):1624–36. doi: 10.1111/cns.13906 (PMC9437226; doi:10.1111/cns.13906)

Full unedited gel/blot for
Figure - 4B TH

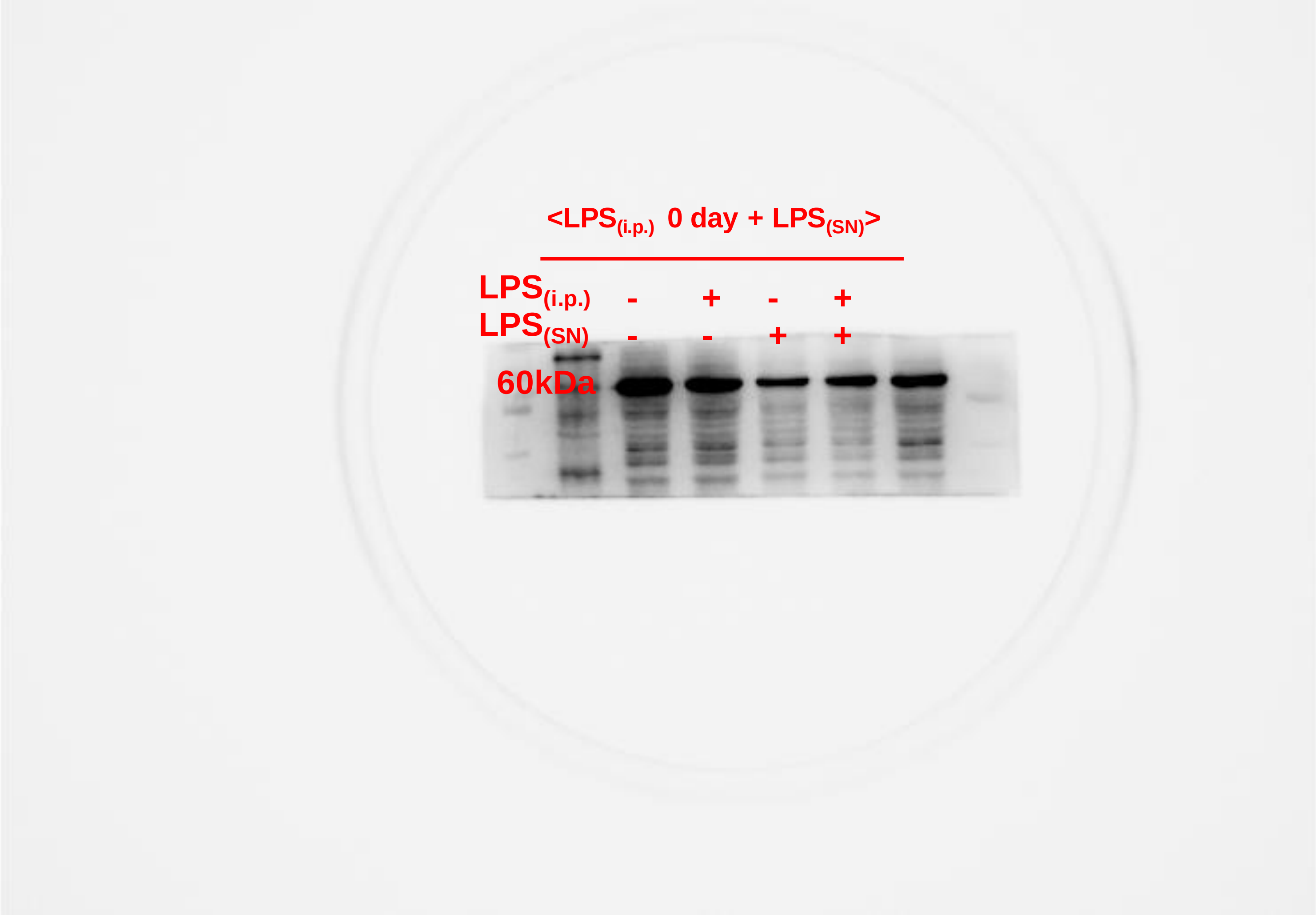

Full unedited gel/blot for
Figure - 4B DAT

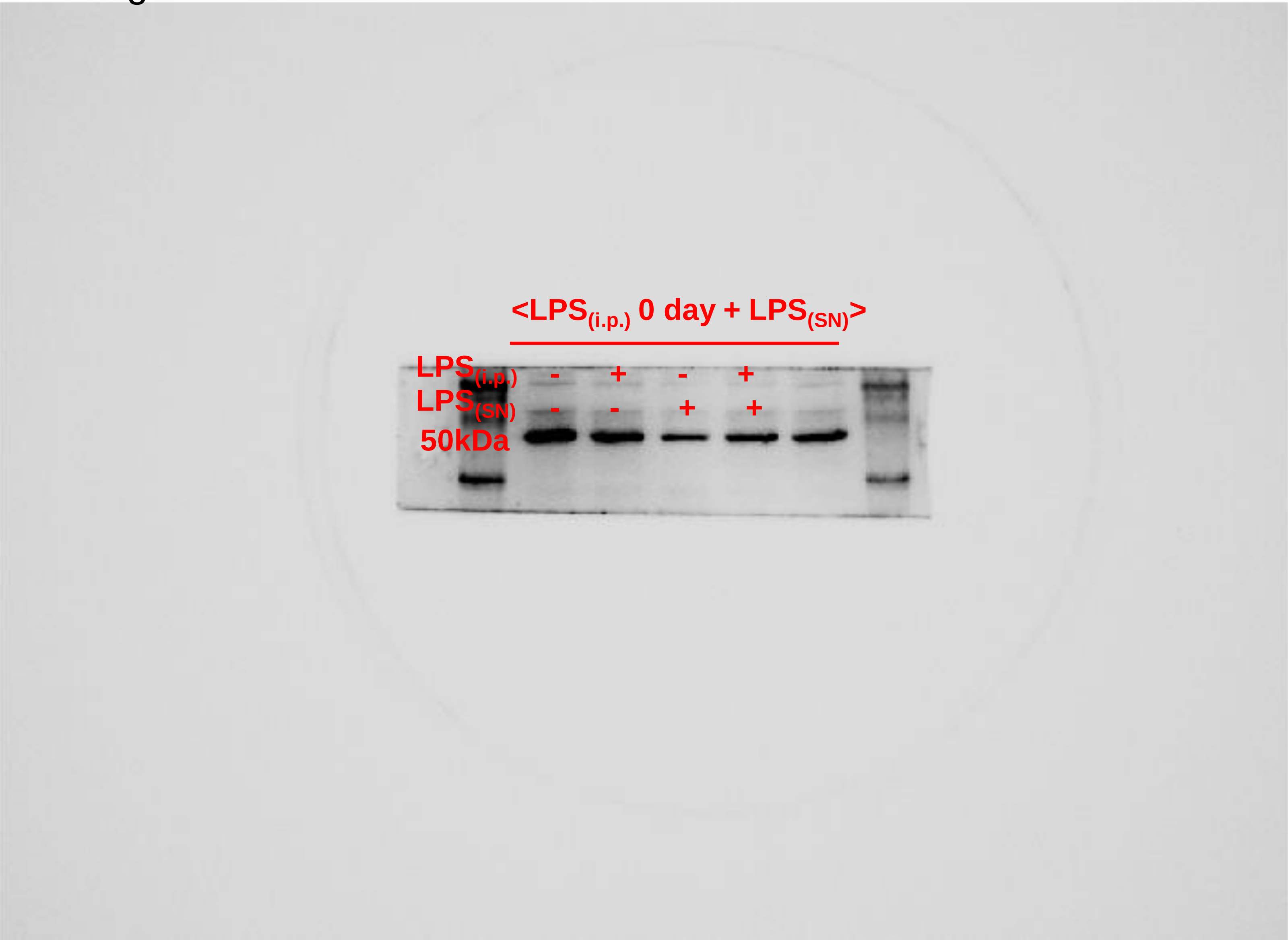

Full unedited gel/blot for
Figure - 4B β-actin

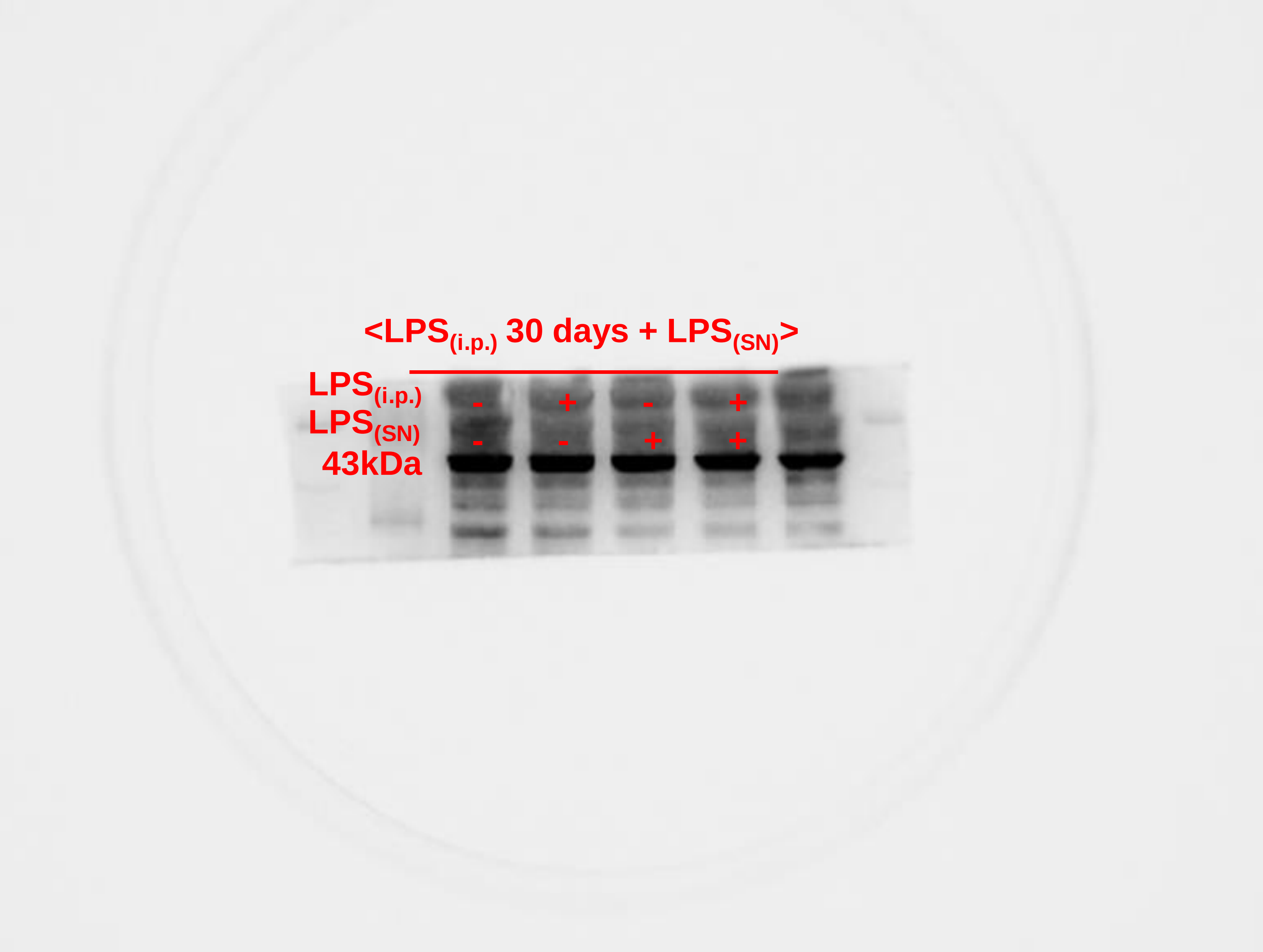

Full unedited gel/blot for
Figure - 4B TH

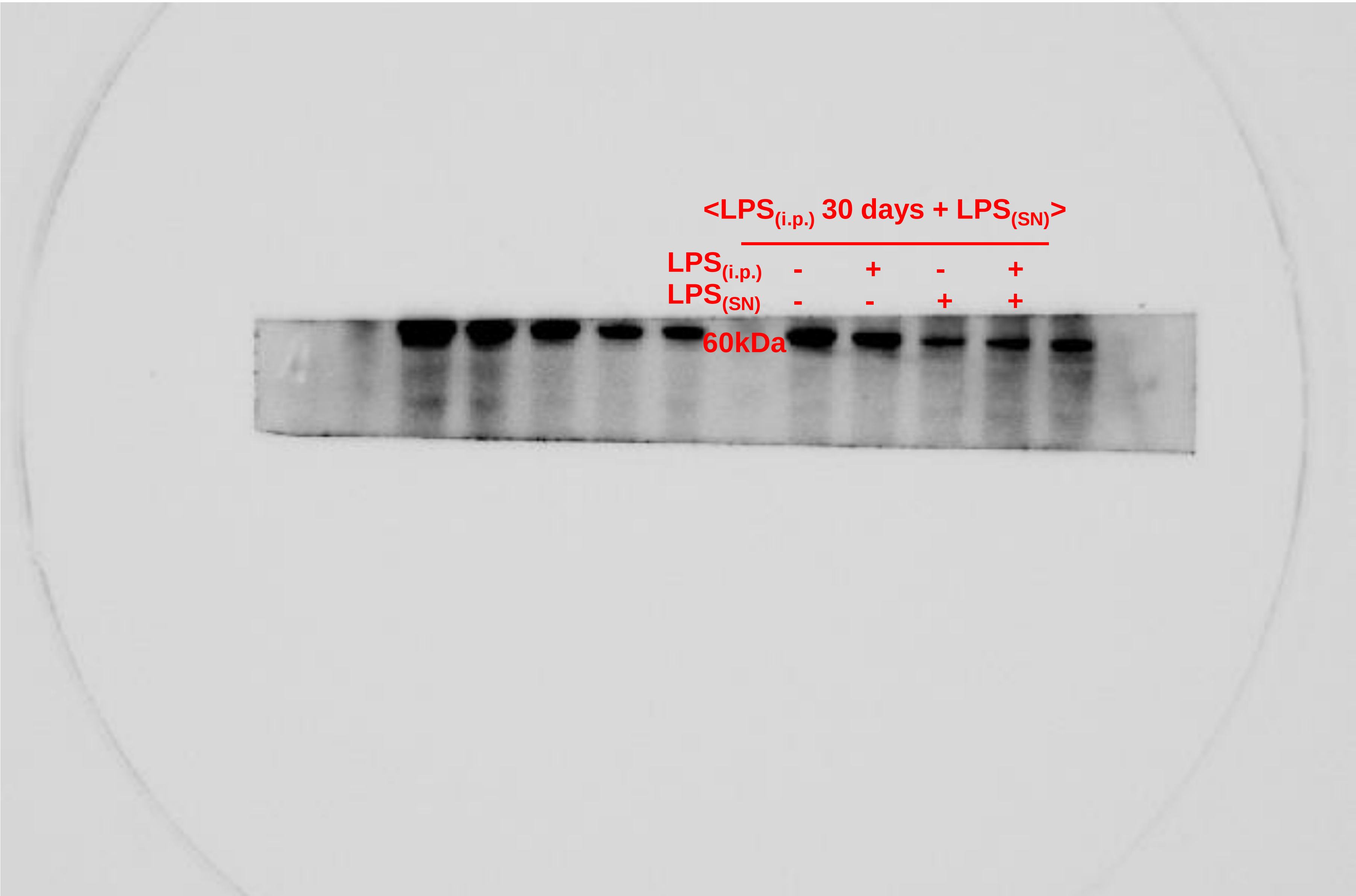

Full unedited gel/blot for
Figure - 4B DAT

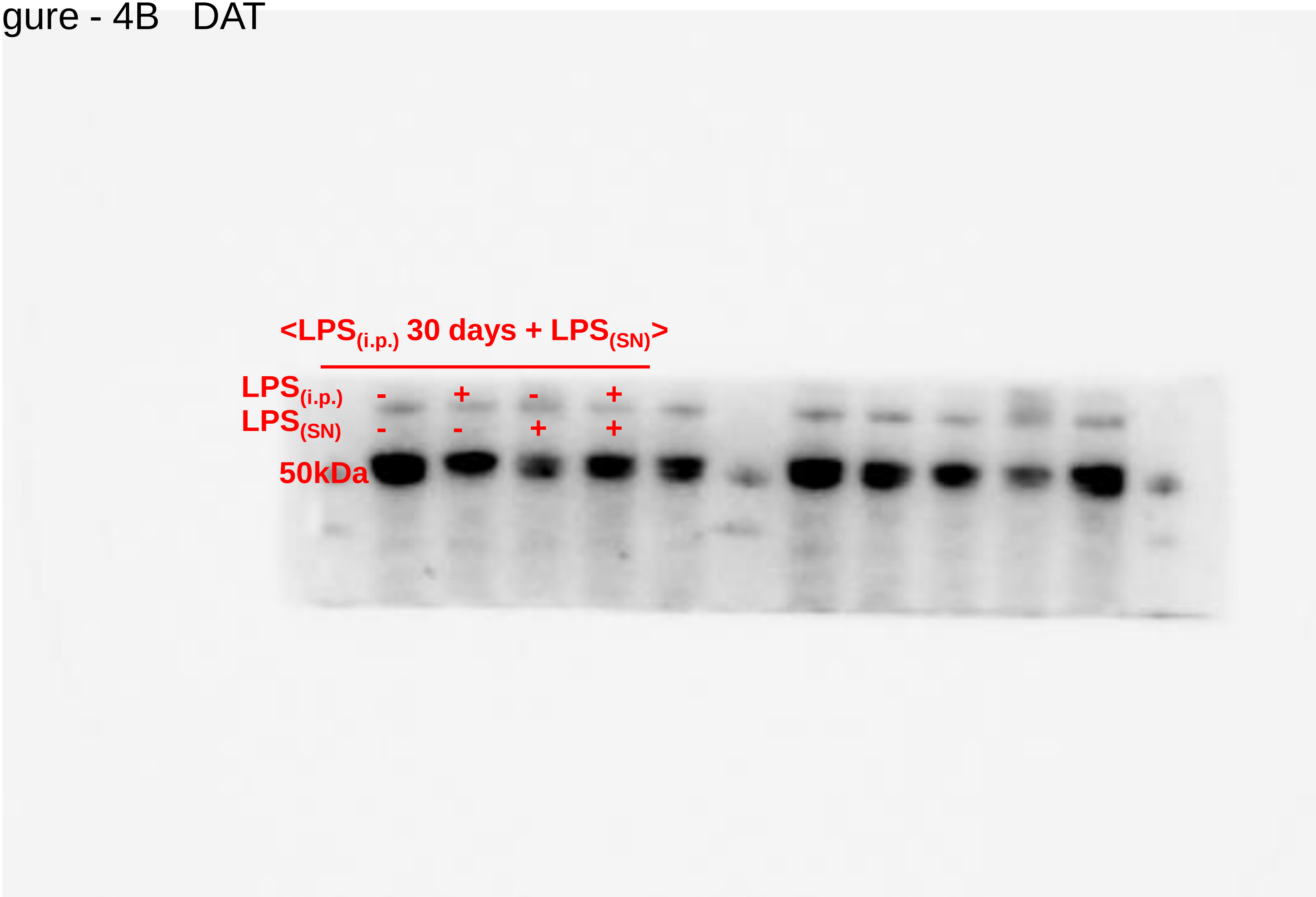

Full unedited gel/blot for
Figure - 4B β-actin

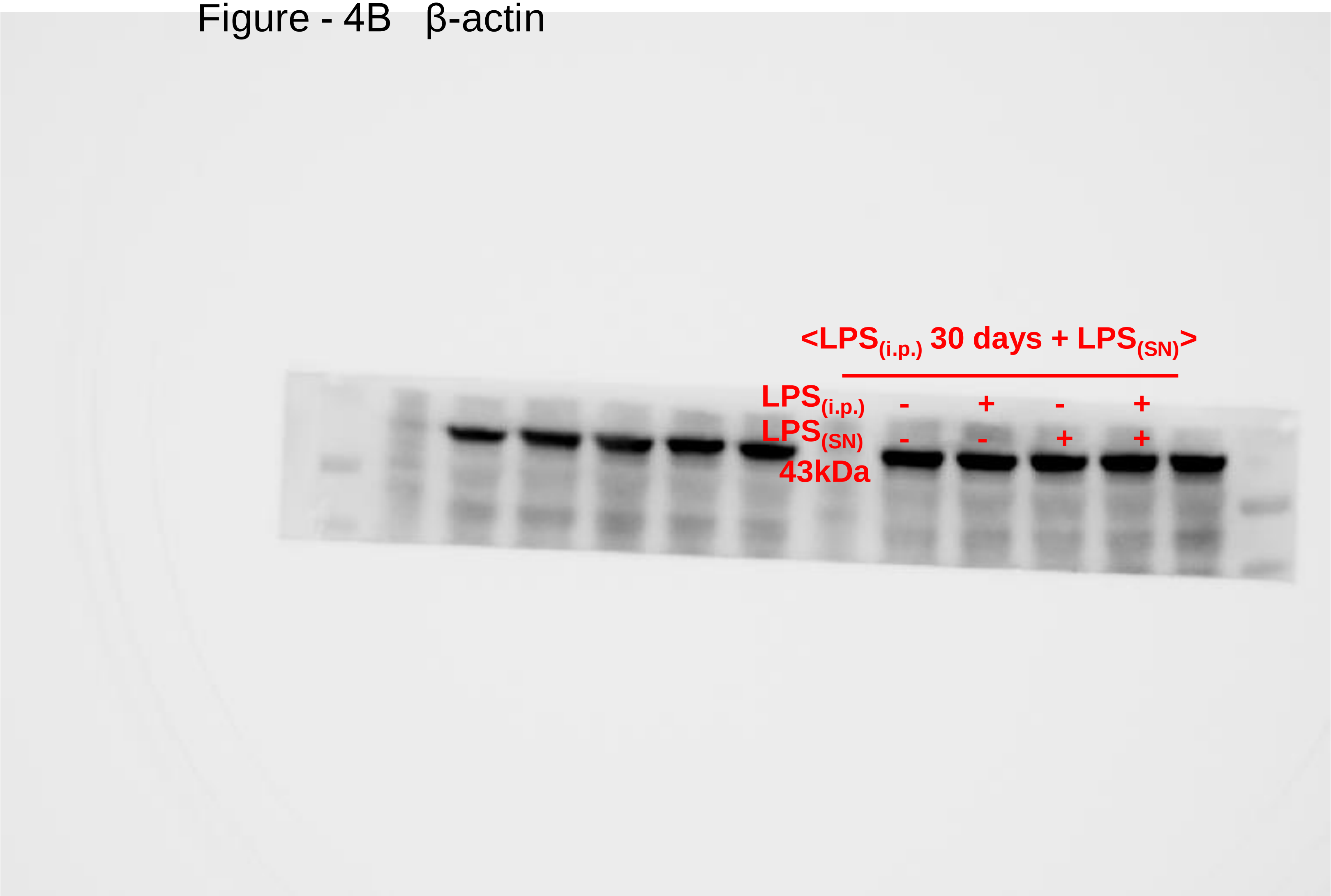

Full unedited gel/blot for
Figure - 4B TH

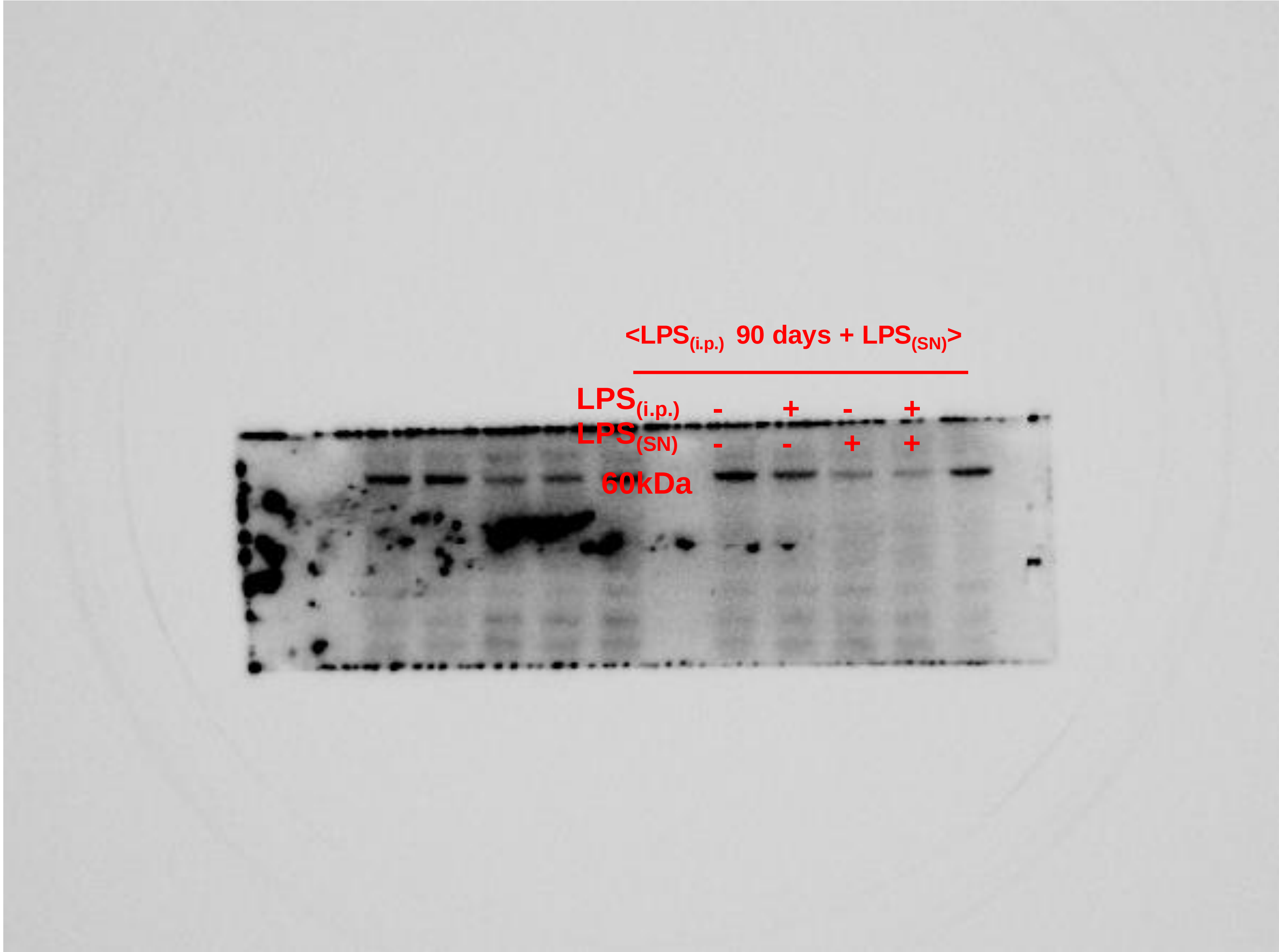

Full unedited gel/blot for
Figure - 4B DAT

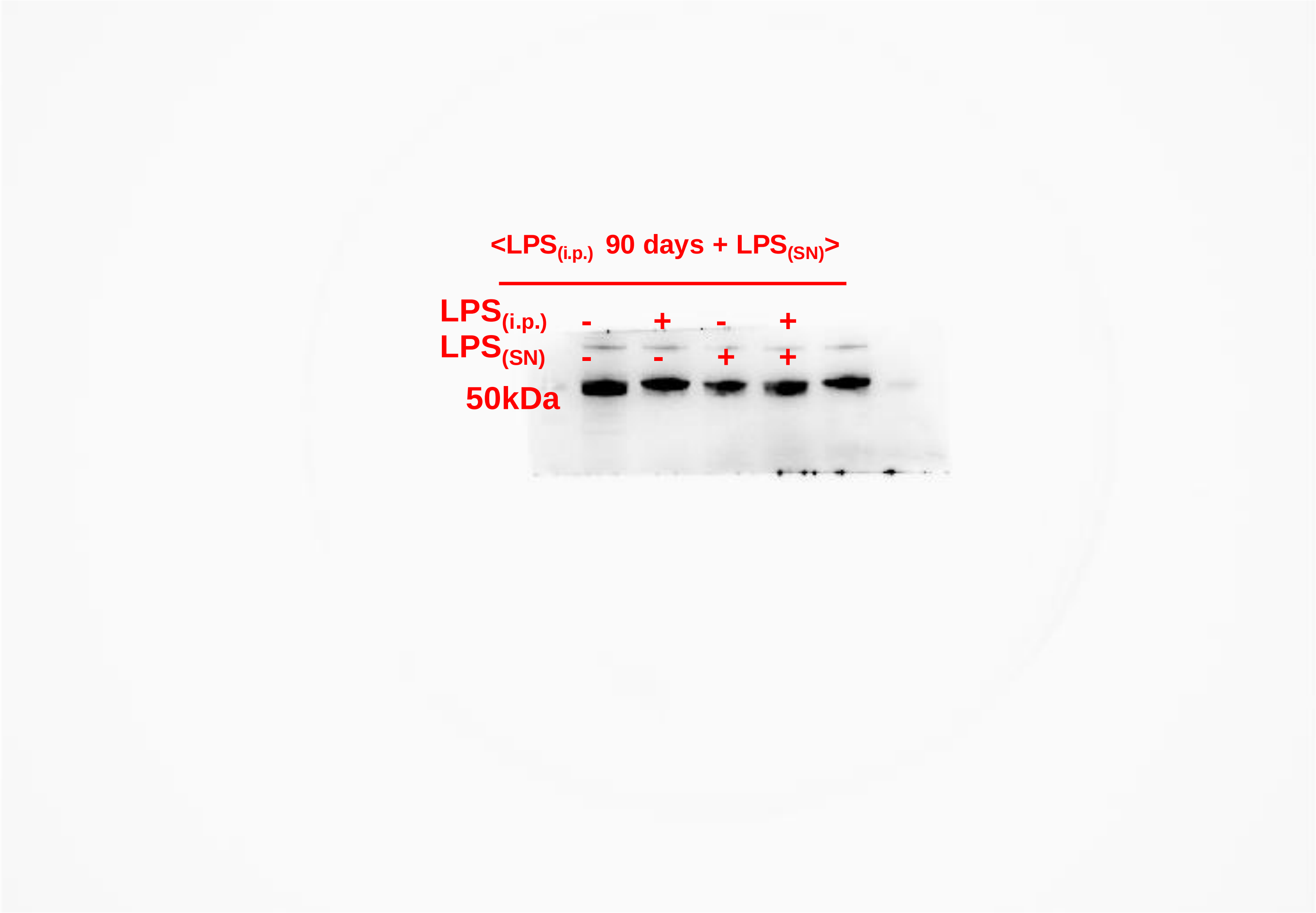

Full unedited gel/blot for
Figure - 4B β-actin

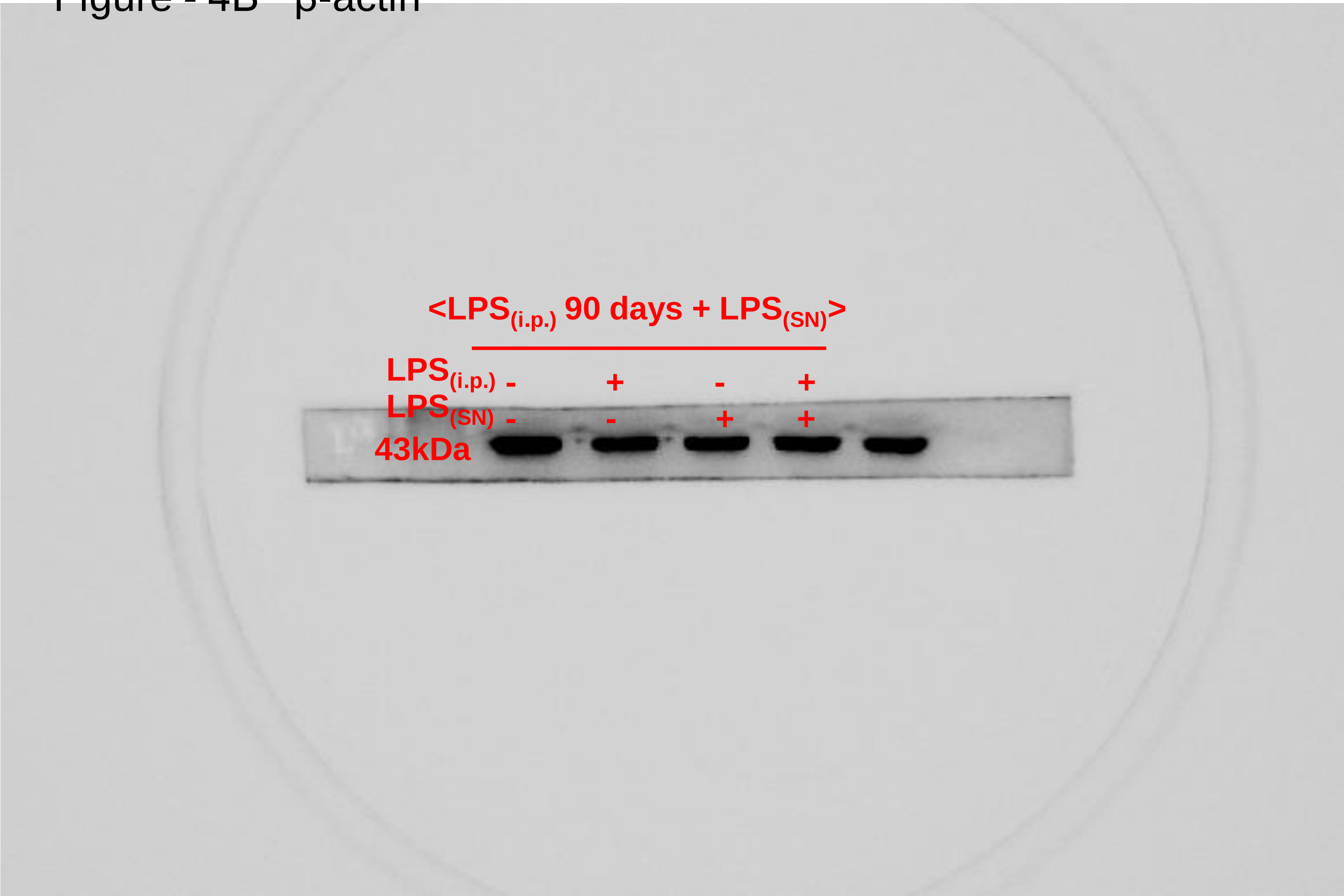

Full unedited gel/blot for
Figure - 5C  IBA-1

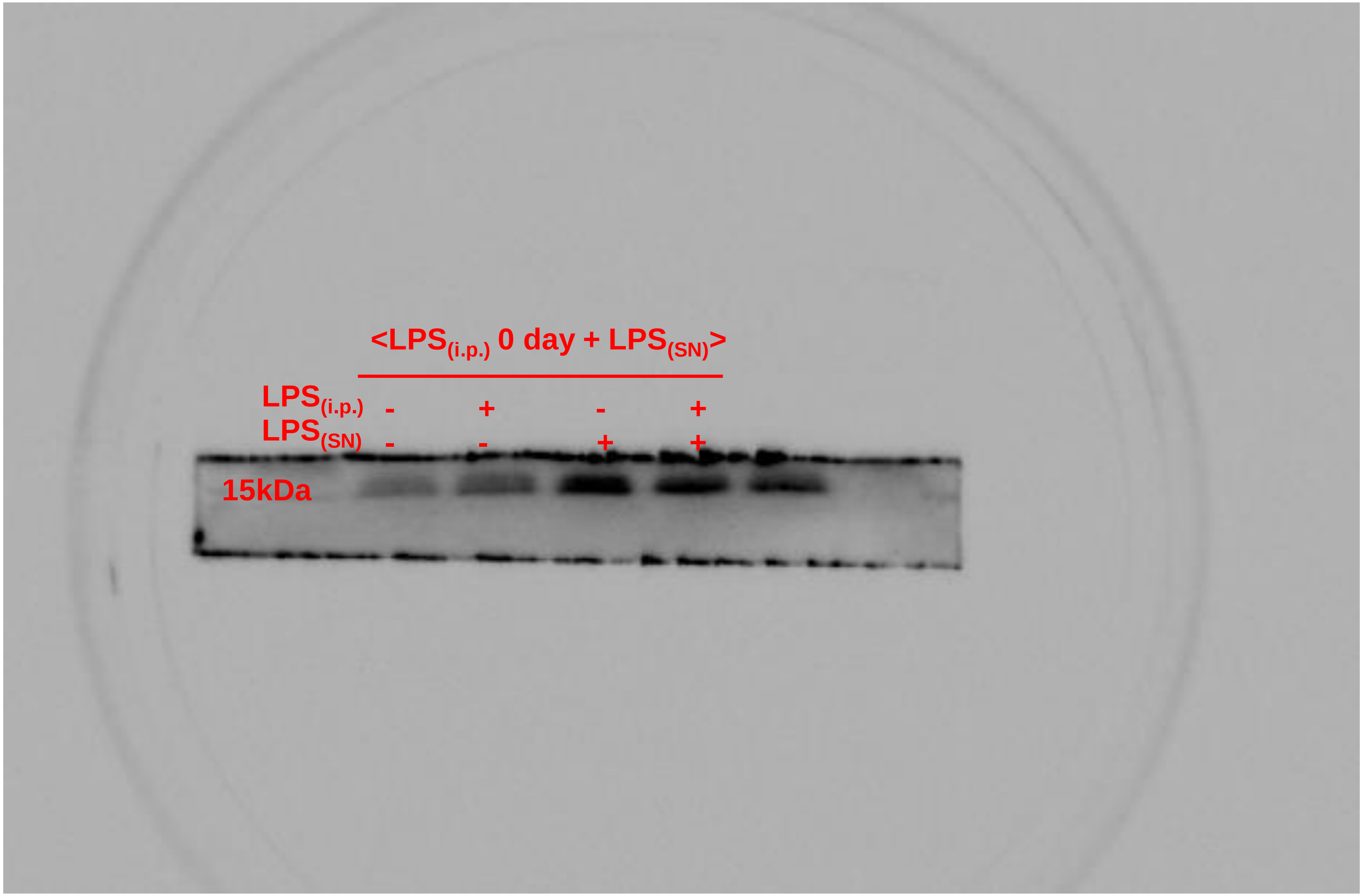

Full unedited gel/blot for
Figure - 5C  GFAP

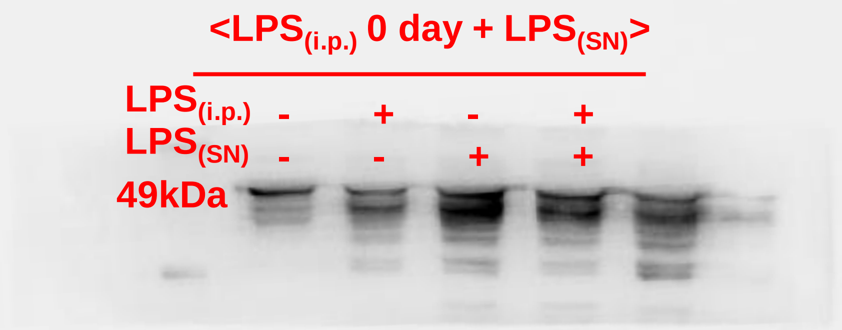

Full unedited gel/blot for
Figure – 5C β-actin

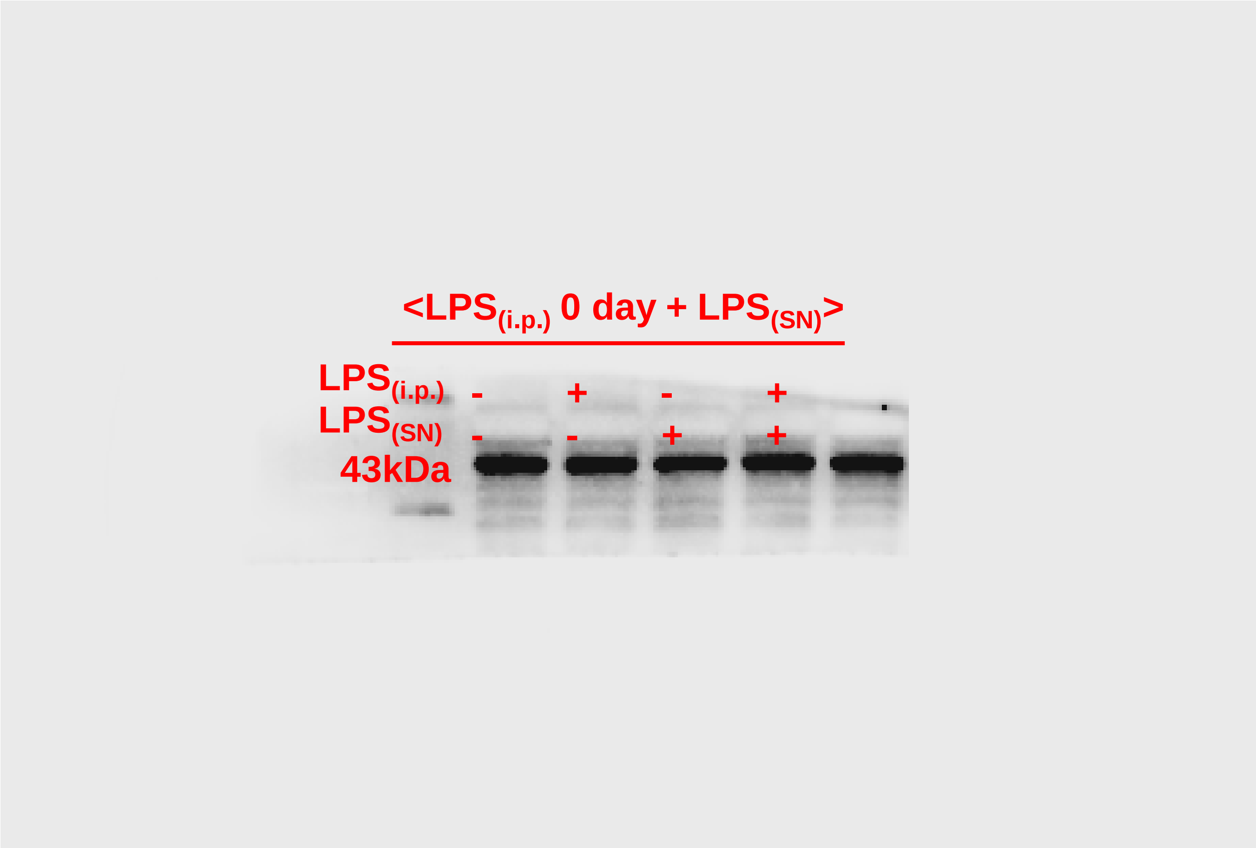

Full unedited gel/blot for
Figure - 5C  IBA-1

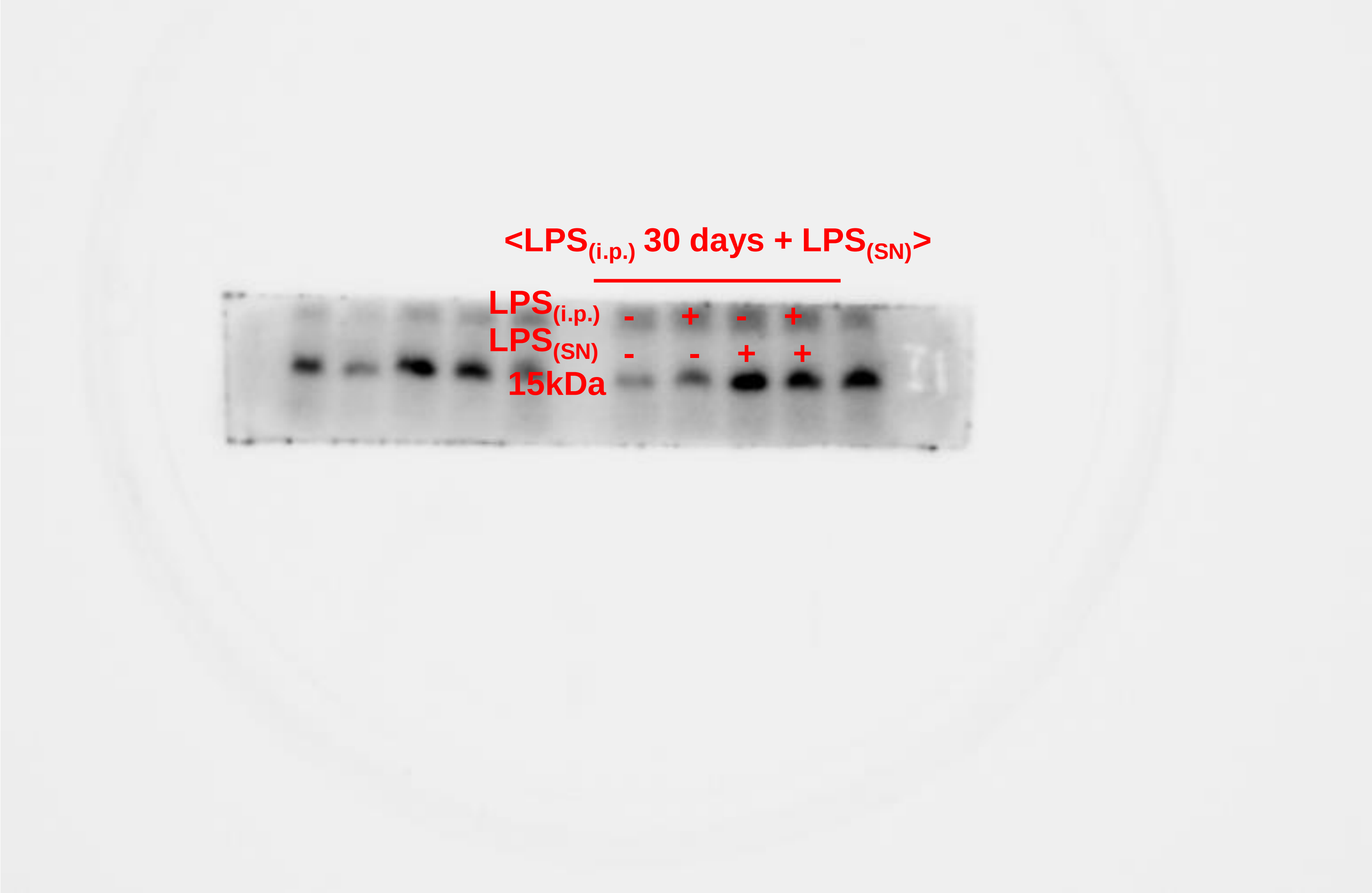

Full unedited gel/blot for
Figure - 5C  GFAP

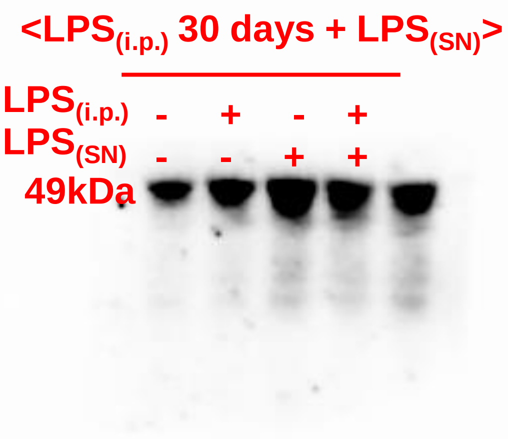

Full unedited gel/blot for
Figure – 5C β-actin

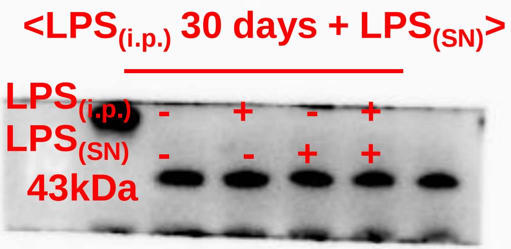

Full unedited gel/blot for
Figure - 5C  IBA-1

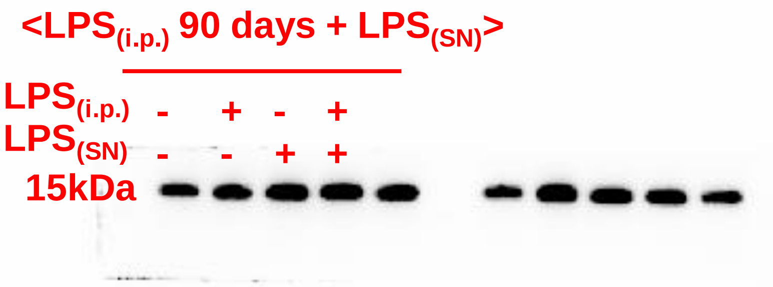

Full unedited gel/blot for
Figure - 5C  GFAP

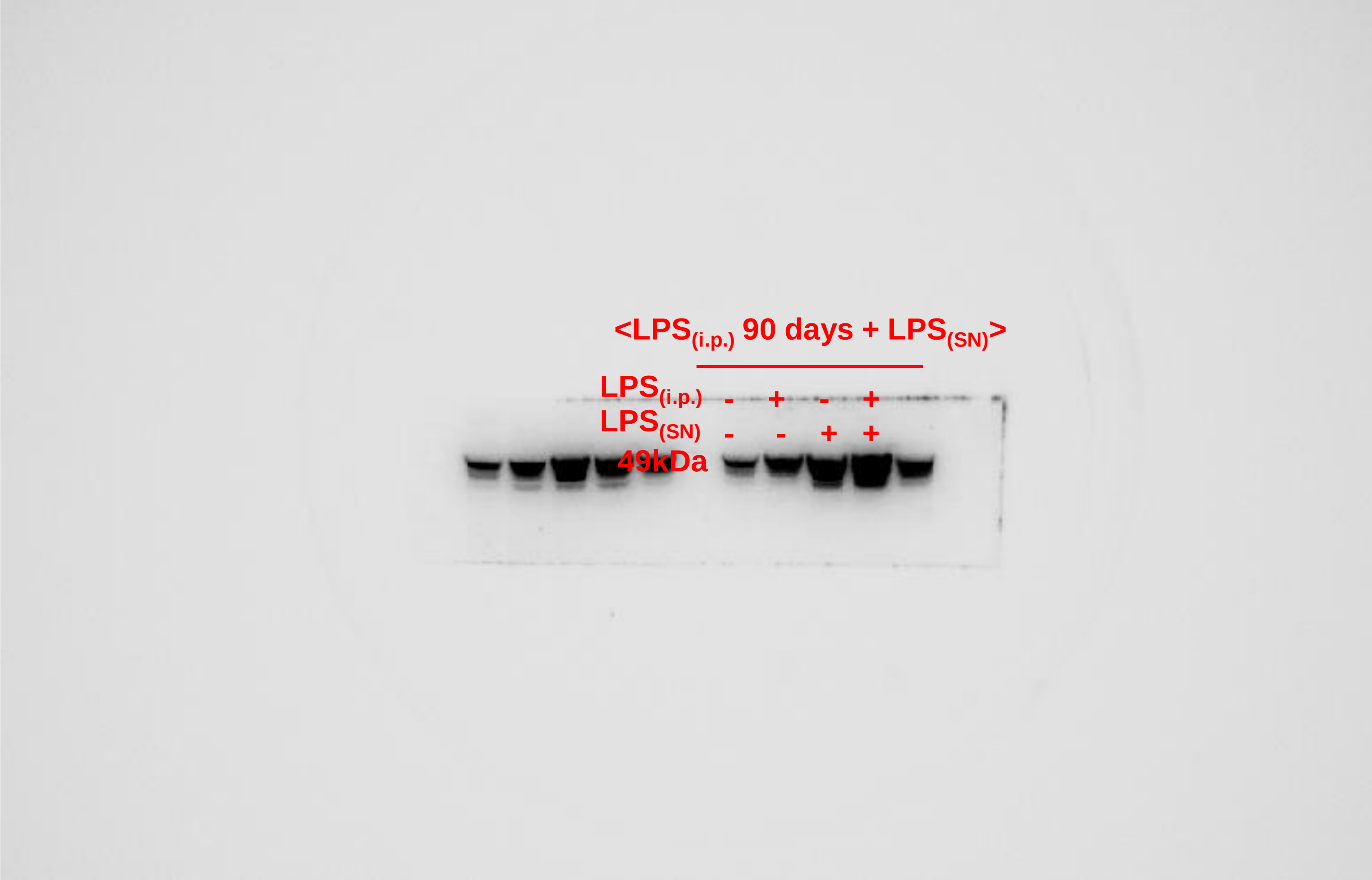

Full unedited gel/blot for
Figure – 5C β-actin

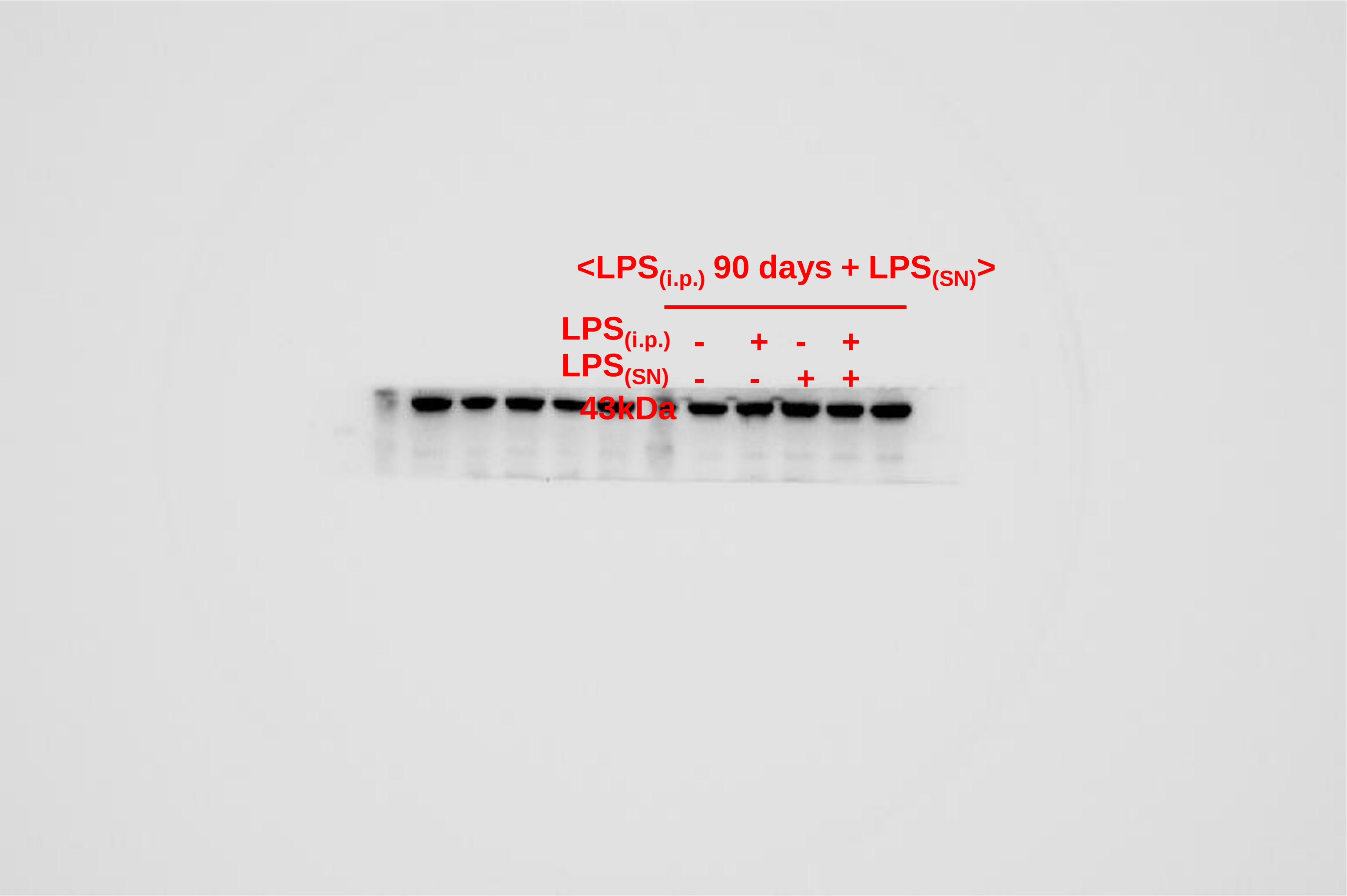

Full unedited gel/blot for
Figure – 6A TNF-α

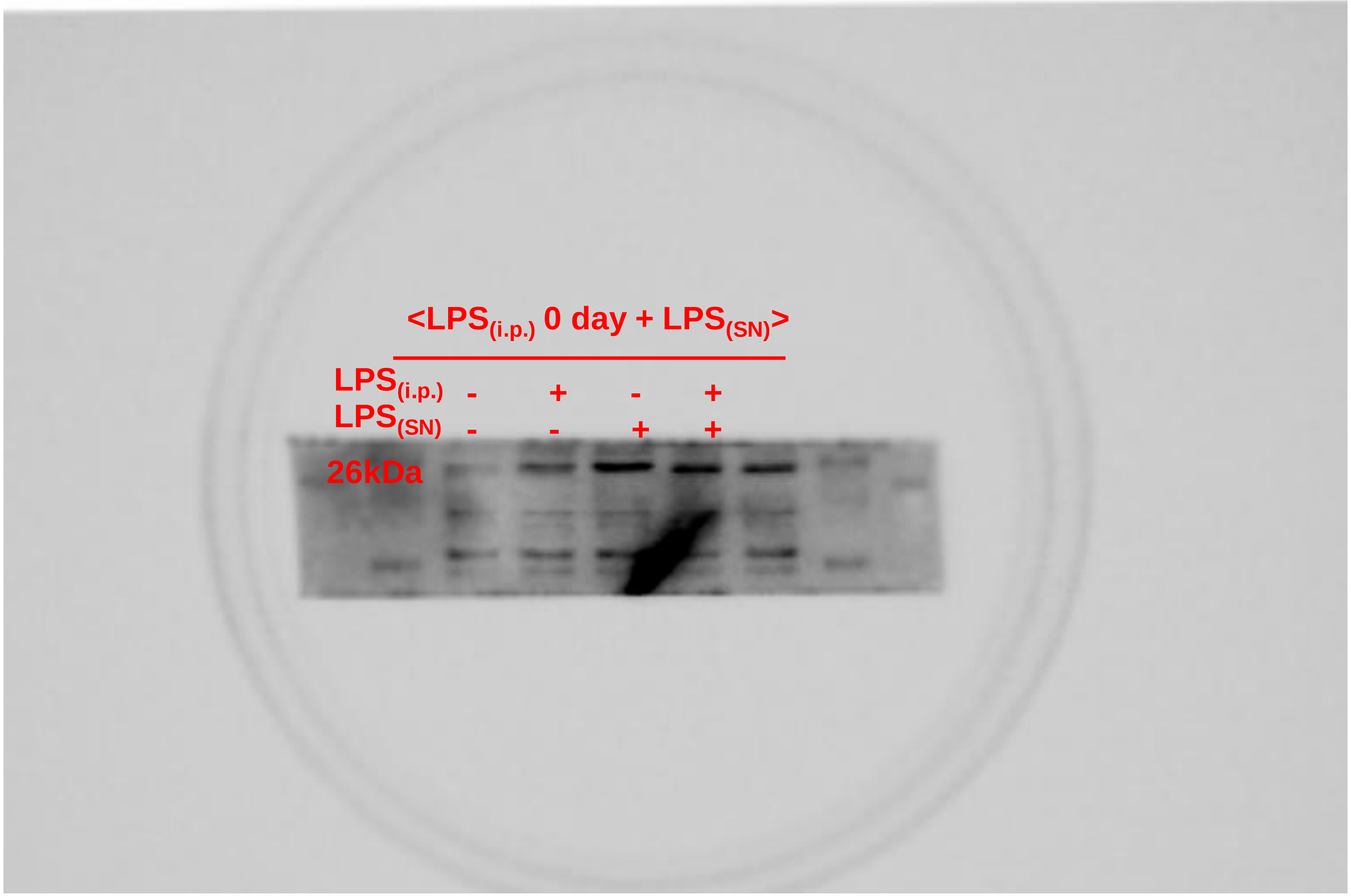

Full unedited gel/blot for
Figure – 6A   IL-1β

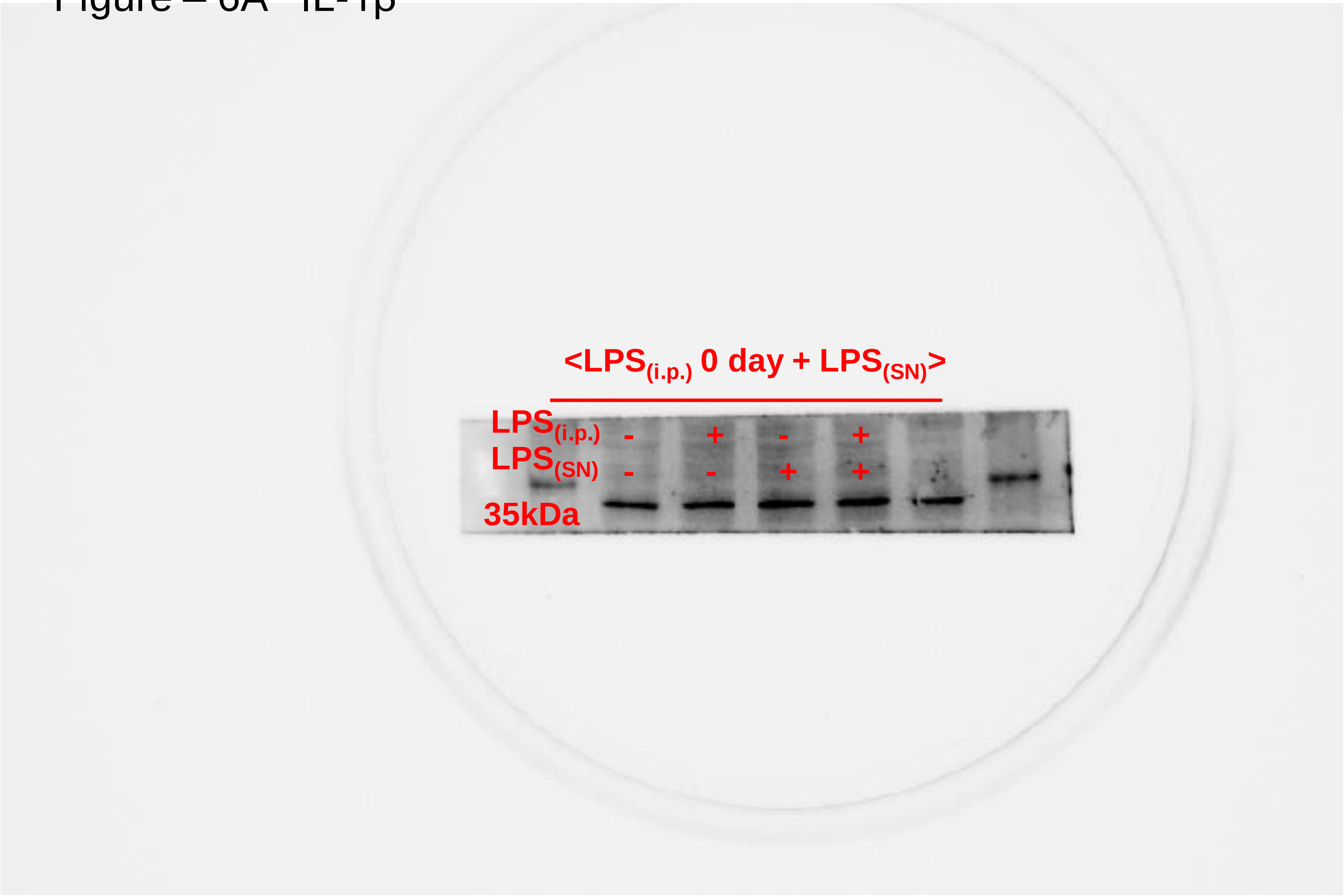

Full unedited gel/blot for
Figure – 6A   β-actin

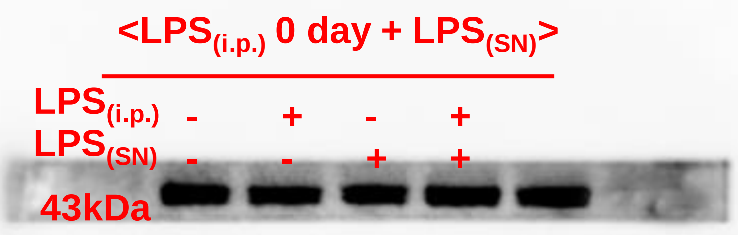

Full unedited gel/blot for
Figure – 6A TNF-α

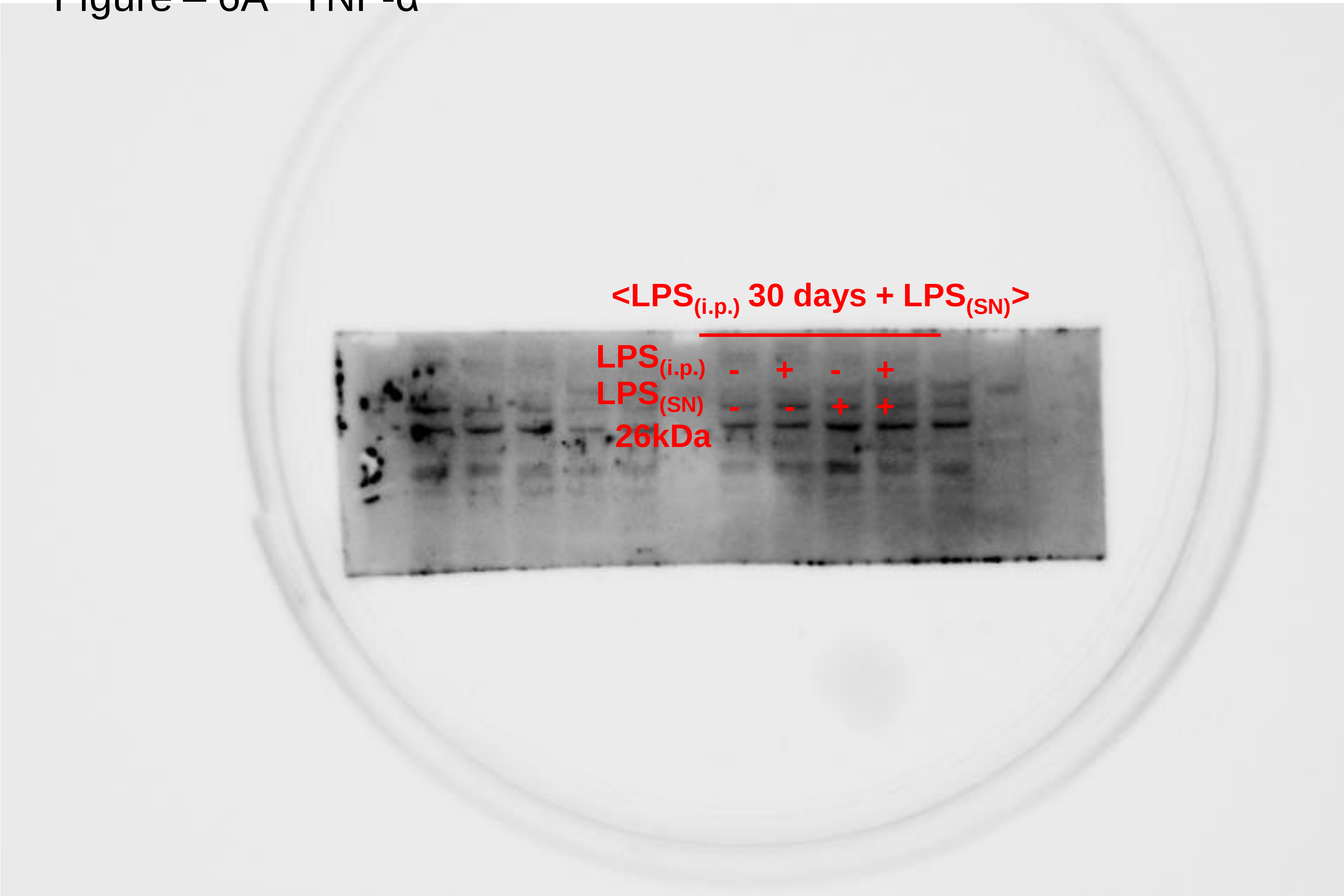

Full unedited gel/blot for
Figure – 6A IL-1β

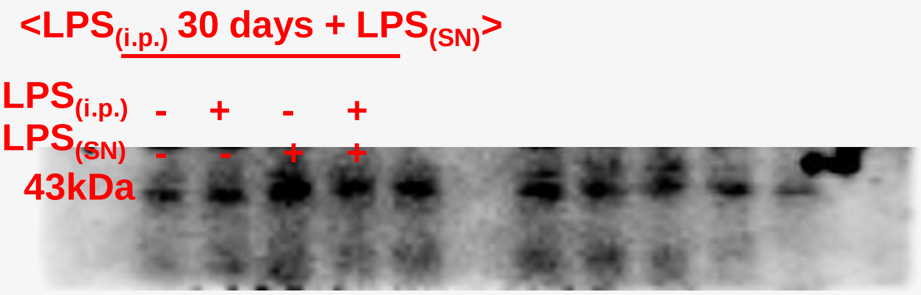

Full unedited gel/blot for
Figure – 6A β-actin

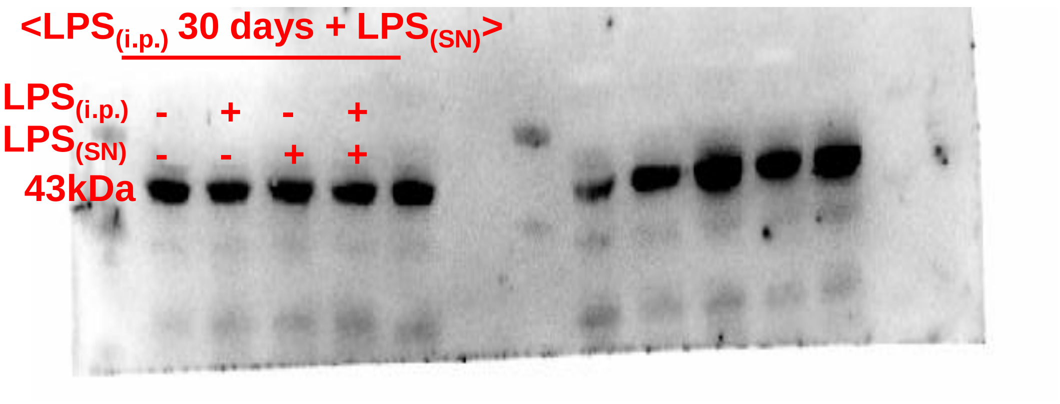

Full unedited gel/blot for
Figure – 6A TNF-α

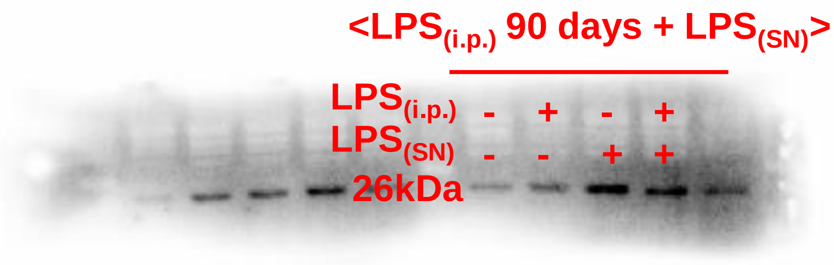

Full unedited gel/blot for
Figure – 6A IL-1β

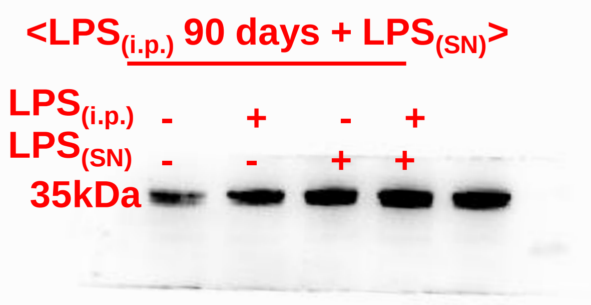

Full unedited gel/blot for
Figure – 6A β-actin

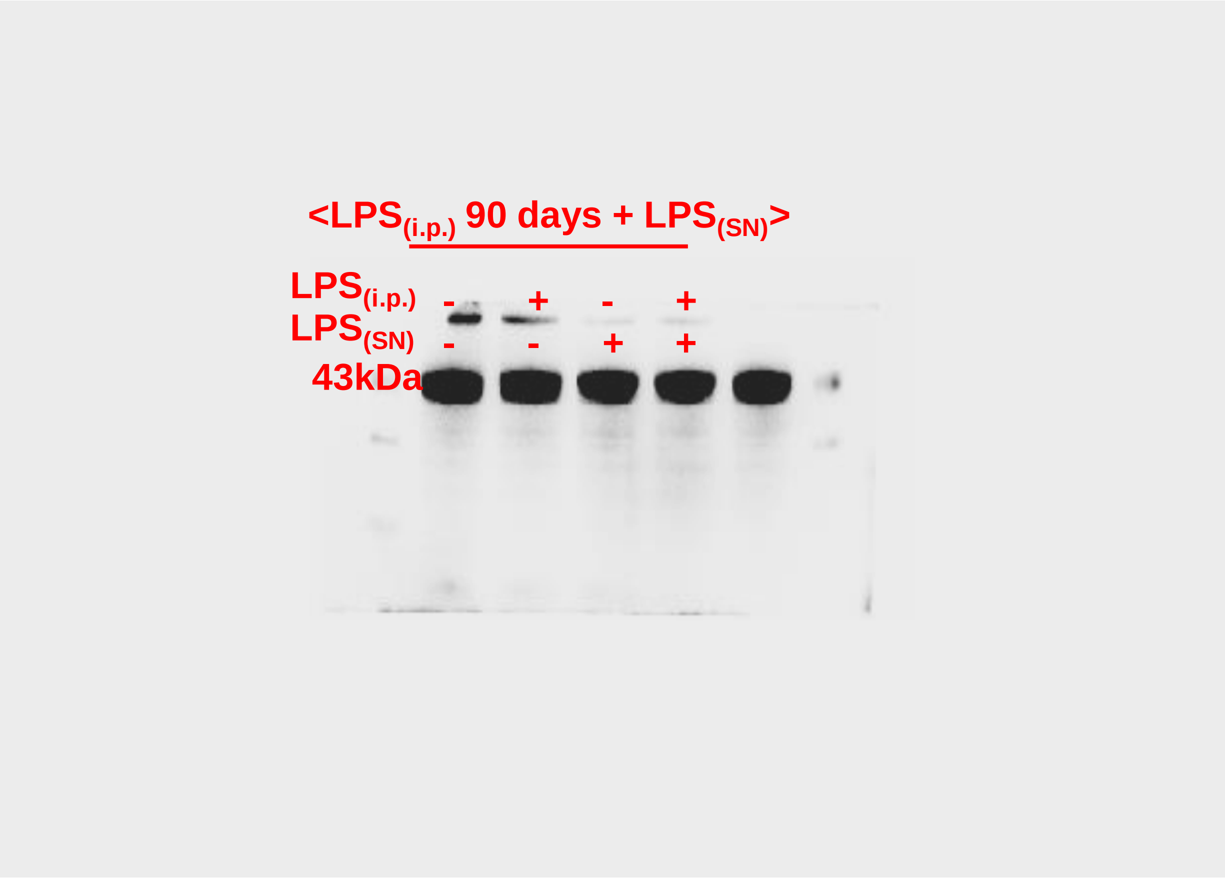

Supplement: Supplementary file 1 — Appendix S1 [file CNS-28-1624-s001.pdf]
